# A Multidisciplinary Hyper-Modeling Scheme in Personalized In Silico Oncology: Coupling Cell Kinetics with Metabolism, Signaling Networks, and Biomechanics as Plug-In Component Models of a Cancer Digital Twin

**DOI:** 10.3390/jpm14050475

**Published:** 2024-04-29

**Authors:** Eleni Kolokotroni, Daniel Abler, Alokendra Ghosh, Eleftheria Tzamali, James Grogan, Eleni Georgiadi, Philippe Büchler, Ravi Radhakrishnan, Helen Byrne, Vangelis Sakkalis, Katerina Nikiforaki, Ioannis Karatzanis, Nigel J. B. McFarlane, Djibril Kaba, Feng Dong, Rainer M. Bohle, Eckart Meese, Norbert Graf, Georgios Stamatakos

**Affiliations:** 1 In Silico Oncology and In Silico Medicine Group, Institute of Communication and Computer Systems, School of Electrical and Computer Engineering, National Technical University of Athens, 157 80 Zografos, Greece; egeorgiadi@uniwa.gr; 2 Department of Oncology, Geneva University Hospitals and University of Geneva, 1205 Geneva, Switzerland; daniel.abler@hcuge.ch; 3 Department of Oncology, Lausanne University Hospital and University of Lausanne, 1011 Lausanne, Switzerland; 4 Department of Chemical and Biomolecular Engineering, Department of Bioengineering, University of Pennsylvania, Philadelphia, PA 19104, USA; aghos@seas.upenn.edu (A.G.); rradhak@seas.upenn.edu (R.R.); 5 Institute of Computer Science, Foundation for Research and Technology—Hellas, 70013 Heraklion, Greece; tzamali@ics.forth.gr (E.T.); sakkalis@ics.forth.gr (V.S.); kat@ics.forth.gr (K.N.); karatzan@ics.forth.gr (I.K.); 6 Irish Centre for High End Computing, University of Galway, H91 TK33 Galway, Ireland; james.grogan@universityofgalway.ie; 7 Biomedical Engineering Department, University of West Attica, 12243 Egaleo, Greece; 8 ARTORG Center, University of Bern, 3010 Bern, Switzerland; philippe.buechler@unibe.ch; 9 Mathematical Institute, University of Oxford, Oxford OX1 2JD, UK; helen.byrne@maths.ox.ac.uk; 10 The Cambridge Crystallographic Data Centre, Cambridge CB2 1EZ, UK; nmcfarlane@ccdc.cam.ac.uk; 11 Department of Computer Science and Technology, University of Bedfordshire, Luton LU1 3JU, UK; dkaba@turing.ac.uk; 12 Department of Computer & Information Sciences, University of Strathclyde, Glasgow G1 1XH, UK; feng.dong@strath.ac.uk; 13 Department of Pathology, Saarland University, 66421 Homburg, Germany; rainer.bohle@uniklinikum-saarland.de; 14 Department of Human Genetics, Saarland University, 66421 Homburg, Germany; eckart.meese@uniklinikum-saarland.de; 15 Department of Paediatric Oncology and Haematology, Saarland University, 66421 Homburg, Germany; norbert.graf@uniklinikum-saarland.de

**Keywords:** in silico medicine, in silico oncology, cancer, hypermodeling, digital twin, virtual twin, computational oncology, Wilms tumor, non-small cell lung cancer

## Abstract

The massive amount of human biological, imaging, and clinical data produced by multiple and diverse sources necessitates integrative modeling approaches able to summarize all this information into answers to specific clinical questions. In this paper, we present a hypermodeling scheme able to combine models of diverse cancer aspects regardless of their underlying method or scale. Describing tissue-scale cancer cell proliferation, biomechanical tumor growth, nutrient transport, genomic-scale aberrant cancer cell metabolism, and cell-signaling pathways that regulate the cellular response to therapy, the hypermodel integrates mutation, miRNA expression, imaging, and clinical data. The constituting hypomodels, as well as their orchestration and links, are described. Two specific cancer types, Wilms tumor (nephroblastoma) and non-small cell lung cancer, are addressed as proof-of-concept study cases. Personalized simulations of the actual anatomy of a patient have been conducted. The hypermodel has also been applied to predict tumor control after radiotherapy and the relationship between tumor proliferative activity and response to neoadjuvant chemotherapy. Our innovative hypermodel holds promise as a digital twin-based clinical decision support system and as the core of future in silico trial platforms, although additional retrospective adaptation and validation are necessary.

## 1. Introduction

Over the last decades, a plethora of mathematical models have been developed, addressing different aspects of tumor complexity ranging from tumor growth to heterogeneity in tumor microenvironment [1,2,3,4,5,6,7,8]. Furthermore, the massive amount of human biological data being produced by multiple and diverse sources (imaging, expression, mutation, clinical, etc.) necessitates the development of integrative approaches able to summarize all this information into answers to specific clinical questions related to treatment responses and prognoses. Herein, we present an integration scheme able to combine component models of diverse cancer aspects regardless of their underlying method or scale. Our goal is to integrate clinical, treatment, imaging, and genomic data with models, stemming from multiple disciplines in a simple but efficient and coherent way to optimise treatment strategy and promote precision medicine in oncology. The hypermodelling scheme allows for complex multi-scale simulations to be broken down into simpler and more manageable models, so-called hypomodels, that can be combined and potentially be replaced by equivalent ones.

Specifically, the hypermodel presented here is orchestrated as a composition of five different physical components, each one representing different aspects of cancer biology at the genome, cellular, and tissue scale. At the heart of the hypermodel lies the Oncosimulator, a tissue-scale model of cancer cell multiplication, cellular response to treatment and spatial tumor expansion/shrinkage based on the notion of discreteevent-discrete state modeling [9]. The Oncosimulator acts as the hypermodel integrator and is linked with a vasculature hypomodel, a biomechanics hypomodel, a cell kill rate focusing molecular hypomodel, and a metabolic network hypomodel. The vasculature hypomodel describes the transport of nutrients in tumors at the tissue scale. It predicts glucose concentration as a function of the three-dimensional spatial distribution of the vessel volume fraction and tumor tissue. The metabolic hypomodel is a sub-cellular component that delineates the aberrant metabolism of cancer cells on a genomic scale. This component utilizes constraint-based methodologies, specifically employing the Flux Balance Analysis method in which cancer cells optimize their growth rates subject to flux balancing constraints and substrate uptake bounds, governed by glucose availability [10]. The molecular component constitutes an integrated cellular framework to model key cell signaling pathways operating at different time scales. Particularly, the ErbB receptor-mediated Ras-MAPK and PI3K/AKT pathway, and the p53-mediated DNA damage-response pathway, are modeled and integrated to predict the kill probability of tumor cells under specific drug combinations or radiation treatment and patient-specific miRNA expression levels. The Biomechanical Simulator (BMS) is a hypomodel for the simulation of bio-mechanical aspects of macroscopic tumor growth. It models the mechanical constraints on tumor growth and informs its growth direction. It relies on the Finite Element Method (FEM) to compute mechanical stresses and strains resulting from tumor growth or shrinkage in a patient-specific anatomy.

The primary emphasis of this paper lies in the conception and development of an integrated modeling approach that distills available information from diverse individualized patient data. At a technical level, the communication between the hypomodels was achieved by exploiting loosely and tightly coupled topologies. In a tightly coupled topology, a feedback loop exists within the model, resulting in certain other models being revisited. To do so, the execution software must maintain some models in a waiting state while others are processing. Conversely, a loosely coupled topology is devoid of cycles, and a model is deemed complete once it has transmitted its information [11]. Details on the technological infrastructure that was developed for the execution of the hypermodel can be found in [12].

The hypermodel was applied in patients with nephroblastoma or Wilms tumor (WT) and non–small-cell lung cancer (NSCLC), addressing clinical questions related to tumor growth and reaction to treatment over time. All data exploited by the present study have been provided following anonymization through the security framework implemented within the CHIC European Commission-funded program (Project FP7-ICT-600841, [13]).

## 2. Materials and Methods

### 2.1. Component Models

#### 2.1.1. The Oncosimulator

The Oncosimulator is a lattice-based, discrete-event, discrete-state approach that models tumor cell population kinetics at a super-cellular and tissue scale, either under free-growth or treatment conditions. Within the framework of CHIC, two instances of the Oncosimulator concept have been implemented: the Lung Oncosimulator, a model of lung tumor response-to-external beam radiotherapy, and the Wilms tumor, WT, (Nephroblastoma) Oncosimulator, a model of Wilms tumor response to preoperative combined chemotherapy treatment of actinomycin and vincristine. The core algorithms of the Lung and WT Oncosimulators have been previously developed by the In Silico Oncology and In Silico Medicine Group, and the implementation details can be found in [9,14]. Herein, the Lung Oncosimulator was algorithmically extended to account for the effect of external beam radiotherapy as described in [15]. To enable communication with the CHIC platform and the exchange of data with the other component models, new code was developed and added according to guidelines [16] in both instances [see also Section 2.2]. Below, basic notions of the modeling approach, common for both Oncosimulators, are briefly presented.

The reconstructed tumor area is represented by a three-dimensional grid of cubic voxels, named Geometrical Cells (GCs). Each GC belonging to the tumor corresponds to a tissue volume of either 1 or 8 mm^3^ and is occupied by an inhomogeneous community of living and dead tumor cells. Specifically, the tumor is assumed to be organized as a hierarchy originating from a type of immature cell with unlimited mitotic capacity. These cells, termed cancer stem cells, may divide either symmetrically, with probability *P_sym_*, to produce two stem cells, or asymmetrically to produce a stem cell and a cell of limited mitotic potential (LIMP) that follows an aberrant differentiation process. LIMP cells are allowed to perform a specific number of divisions, *N_LIMP_*, before entering an irreversible differentiated state (compartment of differentiated -DIFF- cells). Stem and LIMP cells may exist in a cycling or resting, G0, phase. Cycling cancer cells are distributed into four compartments corresponding to the four cell cycle phases (G1, S, G2, M). The withdrawal probability, *P_sleep_*, of cycling cells to a resting phase following mitosis is regulated by the local conditions of nutrient and oxygen supply. Tumor cells are allowed to spend an average time, *T_G0_*, in G0 phase. Afterwards, they re-enter the cell cycle, with probability *P_G0toG1_*, or die via necrosis. The necrotic loss of resting cells is assumed to be caused by nutrient or oxygen deprivation. Stem and LIMP cell categories may die with rate *R_A_* through spontaneous apoptosis. DIFF cells may undergo either apoptosis with rate *R_ADiff_* or necrosis with rate *R_NDiff_*. The time required for apoptotic and necrotic cells to be permanently removed from the tumor bulk is *T_A_* and *T_N_*_,_ respectively.

The Oncosimulator explicitly models chemotherapy and/or radiotherapy. During chemotherapeutic treatment, a fraction of stem and LIMP cells are assumed to undergo lethal damage by the drug(s). These cells follow a rudimentary cell cycle before apoptotic death through a cell cycle phase dictated each time by the mechanism of action of the specific chemotherapeutic agent. The effect of the drug is assumed instantaneous at the time of its administration. In the case of radiation therapy, lethally damaged cells die through a radiation-induced necrotic mechanism. These cells enter a rudimentary cell cycle and die after undergoing a few mitotic divisions. The probability of cells being hit by irradiation depends primarily on the phase they reside. Cell killing by irradiation is described by the Linear Quadratic or LQ Model [17,18]:

S(*D*) = exp [−(*αD* + *βD*^2^)], (1)

where S(*D*) is the surviving fraction after a (uniform) dose *D* (Gy) of radiation to a population of cells. The parameters *α* (alpha) (Gy^−1^) and *β* (beta) (Gy^−2^) are called the radiosensitivity parameters of the LQ model.

The model integrates cytokinetic, metabolic, pharmacokinetic/pharmacodynamic, and mechanical rules to simulate the dynamic behavior of the tumor over time. A computational grid (regular mesh) is used to model the spatial distribution of cells within the tumor. Two mesh scans address the biological aspects (cytokinetics) and the spatial dynamics (tumor expansion/shrinkage) within the simulation. Specially designed algorithms are employed to control the movement of cells within the mesh, defining tumor spatial evolution as described analytically in [9]. In the framework of the hypermodel presented herein, a biomechanical simulator governs the movement of biological cells within the discretized mesh, as detailed in the subsequent sections.

Thorough sensitivity analyses have been conducted and reported in a number of publications [14,19,20,21].

The model is implemented in C++ programming language (version C++14).

#### 2.1.2. The Molecular Hypomodel

The molecular component explicitly models two types of signaling pathways, which are important determinants of tumor cell fate (death and proliferation) and treatment resistance, as well as the interfaces between them: the ErbB receptor-mediated Ras-MAPK and PI3K-AKT pathways, and the TP53-mediated DNA damage-response pathways.

ErbB Receptor-Mediated Ras/Raf/MAPK and PI3K/AKT Pathways: This part of the model has been adapted from Chen et al. [22] after suitable modifications. It is a continuum ordinary differential equations (ODE)-based model with 504 distinct species, 827 elementary mass action-type reactions, and 252 parameters. It consists of all the ErbB family of receptors and a subset of the homodimers and heterodimers. Though the model includes both epidermal growth factor (EGF) and heregulin (HRG) as the growth factors, for simplicity, we only consider the effect of EGF here. The growth factors activate the receptors which, in turn, initialize the downstream signaling cascade. This component model incorporates the effect of receptor internalization and recycling and the cross-talks involved between the Ras-MAPK cascade and PI3K/AKT cascade. This has also been extended to consider various mutant forms of EGFR receptor like L858R and deletion mutants, which can be constitutively active.

TP53-Mediated DNA Damage-Response Module: This part of the model has been adapted and modified from [23]. It consists of two main submodules corresponding to cell cycle progression and apoptosis. The first module consists of cyclin-CDK-mediated cell cycle progression, which affects the G1-to-S phase entry of the cell cycle. The cell death module is the intrinsic apoptotic pathway, which is mediated by key proteins like Bax and Bcl-2, which regulate the cell death protein Caspase 9. Damage to DNA activates ATM kinase, which in turn activates TP53 through kinases Chk1 and Chk2. Activated TP53 in turn activates DNA damage-repair pathways or cell-death pathways depending on the extent of damage. The ultimate cell fate will depend on the combined interactions of all the various components of the network and their initial activation state. This module consists of 16 nodes with 160 negative and 218 positive feedbacks. The module is modeled using a discrete Boolean model. Depending on set thresholds, each node of the network can have two possible states—ON or OFF. The interaction between the nodes is also a discrete number, which can be both positive and negative depending on whether it activates or represses the downstream node. These kinds of discrete models can give two possible outcomes: (a) a point attractor, which is a single steady state where the activation state of all the nodes in the network does not change over successive time steps, and (b) a cyclic attractor that corresponds to a sequence of repeating states (cycle). For the current model, there were three possible outcomes corresponding to three different cell fates: (a) cell-cycle progression (point attractor with high cyclin-G and low p53 activity), (b) apoptosis (point attractor high p53 and high caspase activity), and (c) cell senescence (cyclic attractor with oscillations in p53 and Mdm2).

Module Interfaces and Hybrid Simulator Algorithm: The two modules are able to communicate through the states of the common nodes; these are Erk, Akt, and PTEN. We run the modules sequentially, where the final states of the interface nodes obtained from each module are fed to the other module at the start of each new time step. The ODE states of the common nodes are described by continuous time concentration functions, which are discretized by applying appropriate thresholds before passing them to the Boolean module. The Boolean p53 model will pass the activation fraction of the common nodes to the ODE Ras-MAPK and PI3K/AKT module. We assume that the reactions in the Ras-MAPK and PI3K/AKT module are much faster than the p53 module. This enables us to partially uncouple these processes and pass pseudo-steady state information from the fast to the slow process. We assume that within the short time step of the Ras-MAPK and PI3K/AKT module, the state of the p53 module is invariant. On the other hand, the p53 module will evolve with its own time scale, but its behavior will be modified by the information about the interface species it receives from Ras-MAPK and PI3K/AKT module. The modules are run until the p53 module (slow process) converges to a steady state (point or cyclic attractor).

All model parameters, as well as local and parametric sensitivity analysis, are reported in detail in [24]. Partial clinical validation and acceptance of the molecular models have been performed by comparing the tumor volume data obtained from patients pre- and post-chemotherapeutic treatment against the computed cell kill probability obtained from the patient-specific molecular model [24].

The molecular model is implemented by combining an ordinary differential equations-based continuous-time biochemical network systems model implemented using the COPASI open-source software (version 4.20) and a discrete-time discrete-state Boolean model for transcriptional control of cellular states implemented using the Python programming language (version 2.7).

#### 2.1.3. The Vasculature Hypomodel

The vasculature hypomodel describes the transport of nutrients, glucose in particular, in tumors at the tissue scale. It uses the finite difference method to predict nutrient concentrations as a function of the three-dimensional spatial distribution of vessel volume fraction and tumor tissue, the latter being provided by other hypomodels. The vessel volume fraction is assumed fixed in time. The vasculature hypomodel returns a three-dimensional nutrient field, which can be used as input to other hypomodels.

In general, the vasculature plays a vital role in the transport of nutrients and therapeutics to tumors, with tumor size limited by its ability to co-opt and maintain a vessel network. The vasculature hypomodel is motivated by the well-known model of vascularized tumor growth by Hahnfeldt et al. [25]. This model describes the rate of change of tumor volume T as a function of carrying capacity K, assuming a spherical tumor:

(2) $$T^{\prime }=-\alpha Tlog\left(\frac{T}{K}\right)$$

where α is a growth rate parameter. The adopted rate of change of the carrying capacity term is:

(3) $$K^{\prime }=-\alpha_{2}K+bT-dKT^{\frac{2}{3}}$$

where $\alpha_{2}$, b and d are constants. Implicit in this form is a balance between a rate of increase in carrying capacity due to tumor stimulation of new vasculature, and a rate of decrease in carrying capacity due to increasing diffusion length scales as the tumor grows. Since 3D imaging data and other hypomodels can give a more general tumor shape as inputs to the hypomodel, it is useful to relax the spherical tumor assumption.

**Figure 1:**
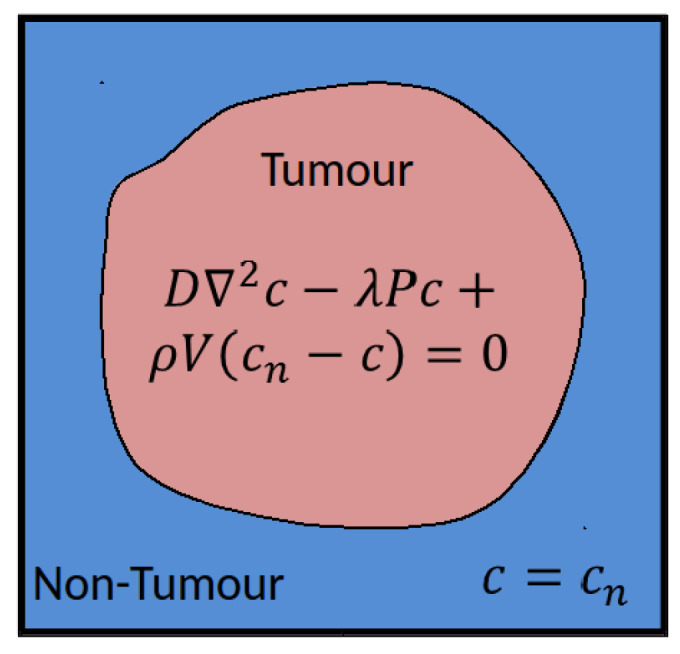
The simulation domain for the transport problem. Distinct tumor and non-tumor regions are assumed.

The vasculature hypomodel assumes steady-state, diffusion-limited transport of nutrients with concentration c, which is supplied by the vasculature at a rate dependent on vessel amount (volume fraction or density) V and is consumed by tumor tissue at a rate proportional to the number or volume fraction of viable cells P. The tissue is assumed to comprise a tumor region and a non-tumor region, as shown in Figure 1.

In practice, these regions are determined based on segmentations of clinical images, performed in a pre-processing step external to the vasculature component in the hypermodel execution. In the non-tumor region, it is assumed that the tissue is well vascularized and nutrient concentrations are set to a reference value $c_{n}$. In the tumor region nutrient transport is described according to:

(4) $$D\nabla^{2}c-\lambda Pc+\rho V\left(c_{n}-c\right)=0$$

where D is the effective nutrient diffusion coefficient in the tumor tissue, λ (redefined) is the rate of nutrient consumption and ρ is rate of nutrient delivery by vessels. In this simple model the nutrient concentration in the tumor will approach the value in the surrounding healthy tissue as the vessel volume fraction increases, or as the rate of consumption by cells decreases.

For the WT and NSCLC hypermodels the Vasculature component describes the transport of glucose and uses a vessel volume fraction that is fixed in time. This simple model was chosen to aid hypermodel integration and validation, as it has a favorably low number of input parameters.

The nutrient transport problem is solved on a regular finite difference grid in 3D. This method was chosen for computational efficiency and to avoid interpolation when used with the grid-based descriptions of cell growth used in other hypomodels.

The model is implemented in the Chaste [26] open-source C++ framework for soft tissue modelling. A custom Chaste build was developed to allow the incorporation of MUSCLE libraries for run-time coupling of hypomodels and also packaging as a standalone executable. The code can be found on GitHub [27]. Validity checks of the vasculature component are presented in Appendix B.

#### 2.1.4. The Metabolic Hypomodel

Normal mammalian cells are exposed to a continuous supply of oxygen, glucose and other nutrients in circulating blood. In normal cells, glucose is taken up by specific transporters and is converted to pyruvate in the cytoplasm through glycolysis generating 2 moles of ATP per glucose. In the presence of sufficient oxygen, pyruvate is then completely oxidized in mitochondria generating additional 36 moles of ATP per glucose. However, when oxygen is insufficient, pyruvate is redirected away from mitochondrial oxidation and is converted to the waste product lactate. In contrast to normal cell metabolism, Warburg’s observations [28,29] showed that cancer cells produce a substantial amount of energy by inefficiently metabolizing glucose to lactate, independent of oxygen availability- a phenomenon termed the Warburg effect or aerobic glycolysis. The exact regulatory mechanisms of tumor metabolism are far from complete. The tumor microenvironment significantly affects the metabolic activity and rewiring, impacting metabolite transporters and glycolytic enzymes. Signaling pathways involving various oncogenes and tumor suppressor genes have been identified to play a role in the altered metabolism [30,31].

To model the metabolic adaptations of highly proliferating human cancer cells, Shlomi et al. [10] employed a genome-scale human metabolic network, comprising 1496 ORFs, 3742 reactions, and 2766 metabolites [32]. They introduced metabolic demands for biomass synthesis required for high proliferation rates, with the flux through biomass serving as the objective function. Additionally, they considered solvent capacity constraints to further restrict the fluxes of metabolic reactions. Their approach successfully replicated several experimentally observed metabolic characteristics during cancer development.

We extend the work of Shlomi et al. [10] and apply a metabolic strategy that allows for near-optimal growth solution, while maximizing lactate secretion. This approach aims to elucidate the high-flux mechanisms leading to a substantial increase in lactate production observed in tumor cells. Sub-optimal growth solutions have been noted to describe the metabolic capabilities of microorganisms under environmental stress and in the absence of sufficient evolutionary pressure [33] indicating that variability around optimal growth is not unexpected for biological systems, including cancer. The lactate maximization strategy is mathematically described as a two-step optimization problem, akin to the Flux Variability Analysis (FVA) method [34], which has been utilized to identify alternate optimal and sub-optimal metabolic states. The lactate maximization strategy employs an iterative procedure to pinpoint the minimal compromise in growth rate necessary to achieve lactate production [35].

Specifically, in the first step, the optimization problem is solved as described in [10] where cells are assumed to maximize their growth rate subject to flux balancing constraints, uptake bounds in the substrate reactions and the solvent capacity constraint. Within these specific constraints, the first problem aims to identify the maximum growth rate. The second optimization problem aims to maximize the lactate production rate. Additionally, it includes the constraint that the growth rate should not be less than a given percentage, *k*, of the optimal growth rate determined in the first problem. The second step is iteratively performed for decreasing values of *k* until a solution is found, as long as the lactate secretion rate remains below a specified tolerance (0.01 umol/mgDW/h). The model provides a solution closer to optimal growth by varying *k* from its maximum to lower values. The model has demonstrated its ability to capture several metabolic phenotypes observed experimentally in cancer. Slight deviations around the optimal growth rate (90–99%) were found to be sufficient for adequate lactate production, with increasing deviations observed at lower glucose uptake bounds.

The metabolic model is implemented using the COBRA Toolbox-1.3.1 in MATLAB (R2008, Natick, MA, USA: The MathWorks Inc.) with GLPK as the linear programming solver.

#### 2.1.5. The Biomechanics Simulator

The Biomechanics Simulator (BMS) aims to predict the mechanical impact of a growing tumor, its so-called “mass-effect”. Rapid cell division and increase of the number of cells in a tissue segment gives rise to mechanical forces that lead to volumetric growth. This tumor-induced strain results in mechanical stresses in the tumor and the surrounding healthy tissue.

Such tumor-induced biomechanical forces shape the tumor environment and are known to affect tumor growth and evolution [36,37], for example by reducing blood perfusion through compression of intratumoral vessels [38]. For certain tumor locations, such as the brain, mass effect is also of direct clinical importance.

BMS is designed to model a tumor’s mass effect and the resulting mechanical stress distribution on macroscopic length scales, i.e., the tissue level. First, tumor-induced strains are computed as spatially varying volumetric growth factor $\epsilon_{growth}(x)$, where x indicates the location in space, based on volumetric considerations and the ratio of local tumor cell concentration to a reference concentration $c_{0}$. Incorporating these strains into a continuum mechanics model of the tumor growth domain then allows the resulting stresses to be computed. We have previously employed this approach for modeling the mechanical stresses resulting from tumor growth [39,40,41,42].

In the CHIC hypermodeling framework, mechanical information is used to identify the most likely growth direction of the tumor. Thus, the role of BMS is to compute tissue stresses resulting from the current tumor cell distribution in each time step. From this information, the direction for tumor growth and shrinkage are computed to inform the redistribution of tumor cells in the next time step.

BMS relies on the Finite Element Method (FEM) to solve the linear-momentum equilibrium equations with a particular mechanical material model and in a patient-specific anatomy. Its usage involves a pre-processing step in which a personalized FE Model of the patient-specific anatomy is created and parametrized, followed by iteratively coupled execution with OS, as described in Section 2.2.3. A custom pre-processing pipeline has been developed to automate the model configuration process, including the assignment of material properties and boundary conditions from simple configuration options. In combination with automatic segmentation tools, this pipeline permits rapid generation of patient-specific FEM models for personalized simulations (Section 2.3.5). FEM model and pre-processing pipeline are implemented using Open-Source libraries and software packages (CGAL, VTK, FEBio).

### 2.2. Hypermodel Coupling Topology

#### 2.2.1. Oncosimulator—Molecular Hypomodel

Cellular intrinsic sensitivity or resistance to treatment is a determinant of treatment outcome. The cell kill rate (*CKR*), i.e., the fraction of tumor cells to be lethally hit by a given therapeutic regimen is an input parameter of the Oncosimulator (Table 1) and it is explicitly computed by the Molecular model based on the molecular profile (e.g., EGFR mutations, miRNA expression data, etc.) of the patient.

**Figure 2:**
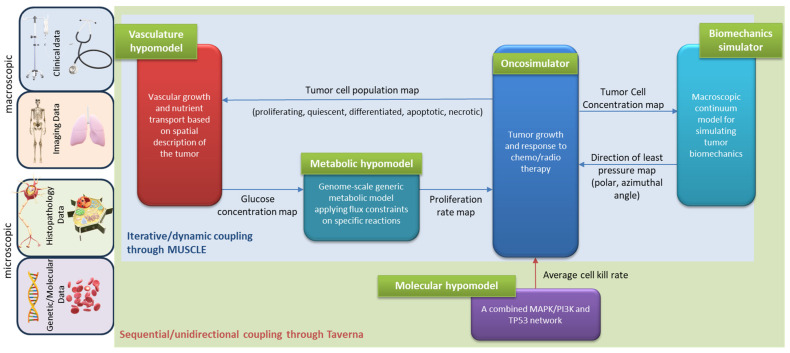
Multimodeler hypermodel communication scheme. The hypermodel contains five hypomodels: oncosimulator (developed by Institute of Communication and Computer Systems), molecular hypomodel (developed by University of Pennsylvania), metabolic hypomodel (developed by Foundation for Research and Technology—Hellas), vasculature hypomodel (developed by University of Oxford) and biomechanics simulator (developed by University of Bern). The arrows represent the flow of information.

The unidirectional data flow between the Oncosimulator and the molecular model has been implemented by a serial coupling topology, i.e., the component models are called and executed sequentially. Within CHIC framework, the aforementioned coupling topology is orchestrated via TAVERNA workflow management system [43] which passes *CKR* as a command line argument to the Oncosimulator (Figure 2).

#### 2.2.2. Oncosimulator—Vasculature—Metabolic Hypomodels

The hyper-modelling scenario dictates an iterative, tightly-coupled communication scheme between the Oncosimulator, Vasculature and Metabolic models (Figure 2).

As tumor grows, well-vascularized regions that provide ample nutrients to cancer cells may coexist with nutrient-limited areas within the tumor mass. In this work, we focus on glucose as the sole limiting resource, although oxygen and glutamine can also be considered. The dependence of glucose uptake on glucose concentration is modeled using Michaelis-Menten kinetics as illustrated in Equation (5). Here, *C* represents the glucose concentration, *V_max_* corresponds to the maximum rate of the process (dependent on factors like GLUT receptor concentration), and *K_m_* is the saturation constant indicating the glucose concentration at which the uptake rate equals to *V_max_*/2. This implies that glucose availability sets an upper limit on the glucose uptake rate. We further assume that *V_bound_* reaches *V_max_* when the glucose concentration *C* reaches its maximum observed value in tissues (*C_max_*= 0.9 kg m^−3^).

(5) $$v_{bound}=v_{max}\frac{C}{K_{m}+C},$$

A relatively slow varying environment is assumed where cancer cells can operate at optimal or near optimal growth rates constrained by the current nutrient availability. The Oncosimulator computes and passes the initial and updated (during execution) tumor domain geometry and population of proliferating, quiescent, terminally differentiated, apoptotic, and necrotic tumor cells at each voxel of the grid (GC-geometrical cell) to the vasculature model. The vasculature model solves the reaction-diffusion equation for glucose transport at each GC, based on the current tumor geometry and cell composition and outputs the normalized glucose field ($\frac{c}{c_{n}}$ at each GC) for use by the metabolic model. The spatiotemporal-dependent inflow of glucose flux is constrained by the Michaelis–Menten kinetics model and an instantaneous optimization problem is solved for each cell position and time point by the metabolic model. During that time interval, the fluxes of the metabolic model are assumed constant. The metabolic model, with the available glucose concentration at every position in the computational grid, provides information regarding the uptake fluxes (e.g., glucose), intake fluxes (e.g., lactate), and the local proliferation rate of the tumor cells that reside within each GC. The latter is passed to the Oncosimulator to update its state.

Considering that Oncosimulator does not have as an intrinsic parameter the proliferation rate of the tumor cells as modulated by local metabolism (see model parameters in Table 1), a parameter transformation needs to take place. The local conditions of nutrient supply, such as glucose concentration, primarily regulate the withdrawal of tumor cells in a quiescent state, in an attempt by the tumor to sustain viability under conditions of reduced nutrient supply [44]. Hence, a reasonable first approximation is to translate the proliferation rate, *a*, of the cell population to the fraction of newborn cells entering quiescent state, *P_sleep_*, within the population. The following formula has been considered:

(6) $$P_{sleep}=\frac{1-e^{aT_{c}}/2}{1-\left.{\left(P\right.}_{G0toG1}/T_{G0}\right)/\left(a+1/\left.T_{G0}\right)\right.},$$

where *T_C_* is the cell cycle duration, *T_G0_* is the residence time of tumor cells in a quiescent state, and *P_G0toG1_* is the fraction of quiescent cells re-entering the cell cycle. Equation (6) has been derived from Equation (7) in [20] that describes the proliferation rate of a tumor cell population with stem and progenitor cell hierarchy as regulated by the symmetric divisions of stem cells, the spontaneous apoptosis, the withdrawal of cells in a quiescent state following mitosis and the cycle entry of quiescent cells:

(7) $$e^{(\alpha +R_{A})T_{C}}=\left(1+P_{sym}\right)\left(1-P_{sleep}+P_{sleep}\frac{P_{G0toG1}/T_{G0}}{R_{A}+1/T_{G0}+\alpha }\right),$$

where *P_sym_* is the fraction of stem cells that divide symmetrically and *R_A_* is the rate of spontaneous apoptosis. As a first approximation, we consider that metabolism has no effect on spontaneous apoptosis and symmetric divisions and, thereby, ignore stem hierarchy and apoptosis by setting *P_sym_* = 1 and *R_A_* = 0. The described parameter transformation has been implemented as intrinsic part of the Oncosimulator.

Furthermore, the proliferation rate, *a*, returned by the metabolic model is an upper bound of the cell cycle (*T_C, max_* = ln(2)/*α*), and corresponds to the case that no newborn cell withdraws to a quiescent state (*P_sleep_* = 0). For cell cycles longer than ln(2)/*α*, the *P_sleep_* computed by (6) is negative and therefore biologically unrealistic.

The previously described cyclic coupling is implemented via MUSCLE platform [16]. The component models are called simultaneously and the data exchanges take place dynamically, i.e., during the runtime of the component models. Data transfer is triggered by the Oncosimulator at every predefined time interval. All the component models run until the Oncosimulation finishes executing. MUSCLE is triggered through TAVERNA.

#### 2.2.3. Oncosimulator—Biomechanics Simulator

**Figure 3:**
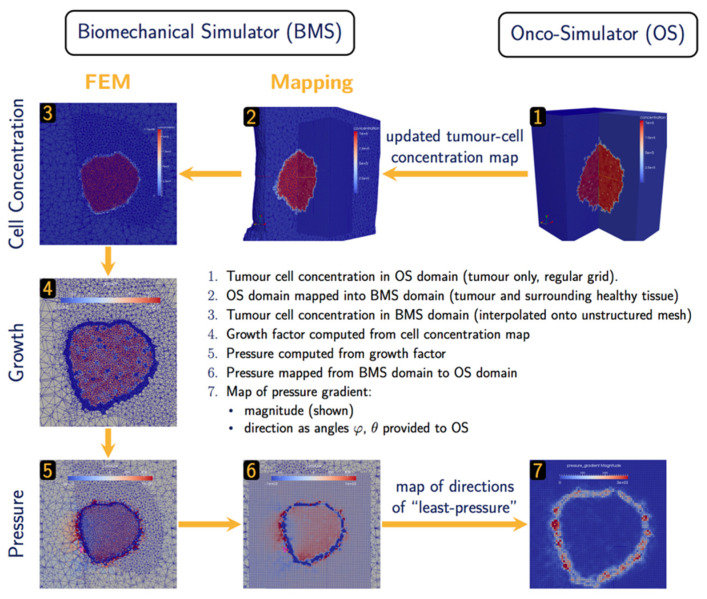
Data exchange and Computation in BMS-OS coupled execution. Arrows indicate the sequence of computational steps: (1) Updated cell concentration map from OS execution; (2–3) Transfer of cell concentration maps from OS to BMS domain; (4) Computation of volumetric growth factor; (5–6) Computation of growth-induced pressure and transfer from BMS to OS domain; (7) Computation of direction of least-pressure in OS domain.

The hyper-modelling scenario dictates an iterative tightly-coupled communication scheme between the Oncosimulator (OS) and the Biomechanics simulator (BMS) (Figure 2 and Figure 3). The dynamic coupling is implemented via MUSCLE as previously described. The Oncosimulator computes cell proliferation in the case of free growth or cell loss in the case of treatment. Starting from an initial spatial map of cancer cell concentrations, BMS receives an (updated) tumor cell concentration map from OS in each time step. BMS and OS operate on distinct domains (OS: tumor only; BMS: tumor and healthy tissue) and discretization (OS: regular grid; BMS: unstructured mesh). Therefore, OS cell concentrations c(x) are first mapped into the BMS simulation domain before spatial maps of local tumor-induced volumetric growth $\epsilon_{growth}(x)$ can be computed. As the predicted mechanical stresses are intended to inform the spatial redistribution of existing tumor cells, we define the “growth” strain relative to a fixed maximum carrying capacity $c_{0}$, below (above) which additional tumor cells may still be accepted (will be rejected) in a region of interest:

(8) $$\epsilon_{growth}(x)={\left(\frac{c\left(x\right)}{c_{0}}\right)}^{\frac{1}{3}}-1,$$

Tumor-induced strain assumes positive (negative) values when the local tumor cell concentration exceeds (is less than) the maximum carrying capacity. From this strain map, tumor-induced mechanical stresses σ(***x***) are computed using a linear-elastic material model with tissue-specific mechanical parameters, Young’s modulus E and Poisson ratio υ. The resulting pressure field $p\left(x\right)=1/3tr(\sigma (x))$ is then mapped back from the unstructured grid of the BMS simulation domain into the regular grid of the OS where the “direction of least-pressure” is computed as the normalized negative pressure gradient:

(9) $$d\left(x\right)=-\frac{\nabla p(x)}{\left\|\nabla p(x)\right\|},$$

This information is returned to OS where it informs the movement and redistribution of tumor cells. Both models and their joint application (not as separate components) have been tested successfully for brain tumor simulation [39].

### 2.3. Tumor- and Patient-Specific Parameterization

The parameterization of component models accounts for the observed interpatient variability for both NSCLC adenocarcinoma and Wilms tumor. By incorporating the molecular information of a patient and by properly adjusting model parameters, a wide range of genetic, anatomical, tumor growth, and tumor response profiles can be reproduced, as described below per component model.

#### 2.3.1. Molecular Hypomodel

Micro-RNAs, short non-coding RNAs that regulate gene expression post-transcriptionally, play a critical role in various forms of cancer. Normalized tissue and serum miRNA expression data of a patient are utilized to adjust the initial levels of the nodes of our networks. In particular, we identify the 5–10 miRNA which are overexpressed in particular patient data. Using miRTarBase [45], we are able to obtain the target proteins of these miRNA. The combined molecular model is run with lower levels of activity or concentration for these target proteins, determined based on the expression levels of miRNA with respect to control. For lung cancer, the model additionally, incorporates the sequencing information by considering the mutational status of EGFR, KRAS, BRAF, and AML/ALK. Hence, the final outcomes are tailored to the particular expression profile of the patients to generate clinically useful outcomes.

For WT, nodes are constrained in a similar fashion, based on the drug interactions. In total, we consider doxorubicin, vincristine, and actinomycin. For each type of chemotherapeutic drug, there exists literature cell kill rates that are uniformly applied for all patients. We aim to obtain an adjusted cell kill rate that takes into account patient-specific genetic variation. To do this, we assume that the effect of the drug on cell survival follows a Poisson distribution so that the fraction of cells killed (*CKR*) is given by $CKR=1-e^{-kt}$ where k is a rate constant that is proportional to the cell kill probability. Using subscripts ‘lit’ and ‘adj’ for literature and adjusted cell kill rates we get $CKR_{adj}=1-e^{-k_{adj}t}$ and $CKR_{lit}=1-e^{-k_{lit}t}$. Then the two *CKR*s are related through the following equation:

(10) $$\frac{k_{adj}}{k_{lit}}=\frac{\ln\left(1-CKR_{adj}\right)}{\ln\left(1-CKR_{lit}\right)},$$

The ratio $\frac{k_{adj}}{k_{lit}}$ is obtained from simulation for a patient and a control where the control indicates no miRNA-based initialization of the model. For a combination of drugs, we assume additivity of rate constants (probabilities) instead of additivities in cell kill rates which is commonly used in literature.

The effect of radiation dosage is implemented through the linear-quadratic model (LQ) [18]. Radiation dosage introduces DNA single and double-strand breaks, which activate the p53-mediated DNA repair and apoptotic pathways. Due to the discrete nature of our p53 mediated DNA damage model, we are only able to account for the dose-independent part (the linear term involving *α*). Future versions will aim to account for the dose-dependent part as well, which requires a more detailed model that takes into account the various double-strand DNA repair mechanisms. In brief, the linear coefficient is used to constrain the node that regulates the p53 activation in a radiation dose-dependent manner. In particular, we activate the ATM kinase levels according to a probability exp(−aD). The survival calculated by the model is then modified by the quadratic term involving *β*. The values of *α* and *β* are obtained from the literature.

Finally, the model is run based on the input miRNA and treatment and averages over several tissue conditions such as growth factor levels and receptor expression. An average as well as a distribution of cell growth, cell senescence and cell kill probabilities are obtained for a given patient. The average cell kill probability is translated to cell kill rate (i.e., fraction of cells killed) that is passed on to the multi-modeler framework.

#### 2.3.2. Oncosimulator

The Oncosimulator accounts for the diverse proliferative behavior observed between tumors based on the balance/interplay between active proliferation, reversible dormancy and cell loss due to differentiation, apoptosis or nutrient/oxygen deprivation. However, there is a lack of patient-specific data directly linked to the related model parameters. Some experimental data may only suggest plausible value ranges. To overcome this limitation, a parametrization methodology has been developed that fits/adapts model predictions to macroscopic clinical proliferation features. We consider that a set of model parameter values constitutes a virtual tumor. The parametrization methodology aims to derive a group of virtual tumors that constitute solutions to the same adaptation problem and efficiently cover the parameter space. These virtual tumors have common a predefined tumor- and patient-specific proliferation profile but, in general, will differ in their treatment response and long-term behavior. The parameter space can be efficiently covered by exploiting computationally low-cost statistical sampling methods, such as Latin hypercube sampling (LHS). If consistent results can be demonstrated for the considered set of virtual tumors (e.g., a clinically significant volume reduction in all cases when applying a specific schedule), this result may be considered robust to the uncertainty in parameter values.

Herein, the proliferation profile of a tumor is described by: (a) the doubling time, *T_d_*, of tumor volume, (b) the growth fraction (*GF*). To perform personalized predictions, *T_d_* can be estimated based on the observed tumor volume increase between two successive imaging examinations, e.g., MRI or CT scans, prior to treatment (S3 in [21]). GF can be determined by immunohistochemistry for the Ki-67 antibody in biopsies or resected specimens. In the case of the non-availability of personalized data, a literature survey can provide biologically reasonable and tumor-specific reference values or value ranges.

Constraints related to other proliferation features of the virtual tumor, i.e., the fraction of stem cells, and necrotic and apoptotic cells, are also imposed. Value ranges of these critical features informed by clinical studies in the literature are utilized.

The parametrization methodology along with the mathematical derivations used to link proliferation features with model parameters are presented in Appendix A. More information can be found in [21,46].

#### 2.3.3. Metabolic Hypomodel

It has been shown that aerobic glycolysis in NSCLC is promoted through oncogenic mutations in two critical proteins, K-RAS and EGFR [47,48]. Ras-driven cancer cells display increased glucose uptake and aerobic glycolysis that support both nucleotide biosynthesis and protein glycosylation for growth signaling. However, it should be noted that high heterogeneity in metabolism proteome has been observed (i) compared to normal lung tissue, (ii) between lung subtypes and (iii) between primary and metastatic lung cancer.

In order to construct a tumor-specific metabolic model in a simplified manner, we included constraints in the metabolic reactions of the model, which are associated with bibliographically reported differentially expressed metabolic genes/proteins in these tumors. mRNA levels cannot accurately determine enzyme concentrations as inaccuracies in experiments, post-translational modifications, and other effects might occur. However, they can determine an upper bound on the amount of available enzyme concentrations. In particular, enzyme levels, *E_i_*, bound the fluxes of the corresponding metabolic reactions *v_i_* through *v_i_* = *Kcat_i_ E_i_*, where *Kcat_i_* corresponds to the enzyme’s turnover number. However, in the absence of quantitative information, metabolic reactions catalyzed by up-regulated metabolic proteins/enzymes, are constrained to carry non-zero fluxes via a lower bound, which is set equal to 0.1 umol/mgDW/h unless stated otherwise, for all the involved reactions. Different bounds have also been tested. It is important to mention that the level of flux bound substantially alters the metabolic capabilities of the cells. Downregulated genes constrain the corresponding reactions via an upper bound, which is usually set equal to zero unless stated otherwise. Parameter values are shown in Table 2.

An extensive omics analysis [47] integrating DNA, RNA, and proteomics data from normal lung, patient primary tumors, and primary tumor-derived xenograft tumors revealed sets of proteins that are consistently up- or downregulated across tumors, recapitulated in xenograft tumors and their associated genes map into regions of focal amplification or deletion respectively. This DNA->RNA->protein association indicates a response to selective pressure driving cancer phenotype. From the reported metabolism proteome clusters in [47], we used specific clusters of proteins (Table 3) consistently upregulated in LADC (cluster index: C15) and LSCC (cluster index: C10) to constrain the corresponding metabolic fluxes of the genome-scale metabolic network. It is also important to mention that individual proteome clusters have been correlated with overall survival in cancers other than NSCLC.

WT is believed to arise from the malignant transformation of renal stem cells that abnormally persist after embryogenesis and maintain embryonic differentiation capacity. Although there are a few studies that have shown metabolic alteration related to glycolytic phenotype in WT, unfortunately, there are no thorough studies currently available, which have investigated the metabolism of WT in detail. There is only indirect evidence from the genes altered in WT that there are alterations in cell metabolism. Thus, in the absence of bibliographic or other data, we use the generic cancer metabolic model to describe WT metabolism, which can be supported by the fact that Wilms’ tumor cells are believed to derive from pluripotent embryonic renal precursor cells.

**Figure 4:**
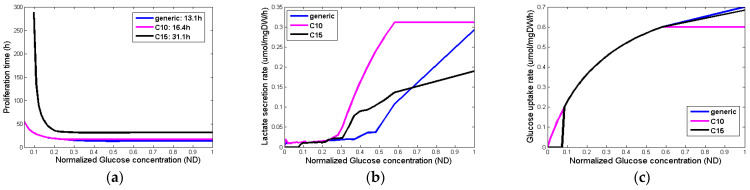
The metabolic capabilities of the generic phenotype (blue line), the C10-constrained phenotype (purple line), and the C15-constrained phenotype (black line) with respect to their proliferation rate (**a**), lactate production (**b**), and glucose uptake rate (**c**) for different (normalized) glucose concentrations. Predicted linking variables between the metabolic model and the Oncosimulator for specific flux bounds in the corresponding reactions.

Figure 4 summarizes the output variables for different glucose concentrations and different phenotypes. Although the glucose uptake rates (Figure 4c) are very similar among the different phenotypes, their proliferation time (Figure 4a), as well as the lactate production (Figure 4b), are substantially different for the different concentrations of glucose.

#### 2.3.4. Vasculature Hypomodel

Although the model has a physical basis, determination and physical interpretation of parameter values (D, λ, ρ, V, $c_{n}$) is challenging. In practice, these parameters should be treated in a phenomenological manner and used in fitting model predictions to observed clinical tumor growth (or shrinkage) rates. Discrimination of the relative influence of delivery by the vasculature and consumption by cells may be aided by additional observations of tumor micro-vessel density and vascular function through functional imaging or cell numbers by functional imaging. However, this has not been performed to date.

In order to obtain reasonable estimates for these parameters for model evaluation, literature values based on observations in tumor spheroids are adopted [50]. Within the hypermodeling framework, dependent components use glucose concentrations to predict cell proliferation rates in the tumor. Although the mechanism of glucose consumption by cells also depends on oxygen availability [50], to preserve the simplicity of the model it is assumed that the diffusing nutrient is glucose and glucose consumption is independent of oxygen concentration. Parameter values are shown in Table 4.

#### 2.3.5. Biomechanics Simulator

The mechanical response evoked by a growing tumor depends on its mechanical growth environment, which is defined by the surrounding healthy tissues, the tumor’s shape, and location, as well as mechanical constraints. The BMS simulator component supports two levels of parameterization to represent (1) different simulation scenarios, such as tumors growing at different body sites, and (2) the patient-specific geometry in any of these scenarios.

In each tumor-growth scenario, we identify those tissues that are expected to make a distinct contribution to the tumor’s mechanical landscape, either because of their immediate vicinity to the developing tumor or because of their distinctly different mechanical properties. Average bulk values are assumed for other tissues. As mechanical boundary conditions, we assume the movement of nodes on the outer surface of the BMS simulation domain to be fully constrained. In both hypermodeling scenarios, the BMS simulation domains are chosen to be significantly larger than the actual tumor growth domain (provided by OS). Scenario-specific tissue types and mechanical properties are listed in Table 5 for the WT and the NSCLC scenarios.

**Figure 5:**
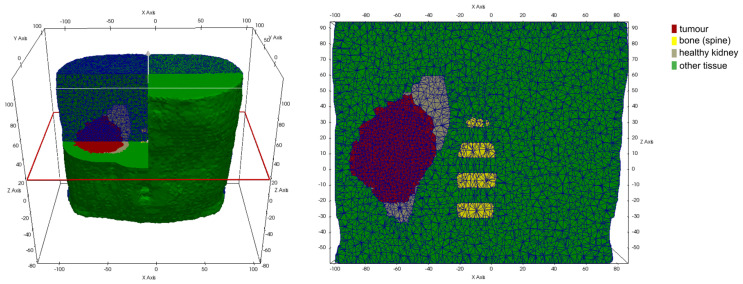
Personalised FEM model for WT scenario with patient-specific FEM simulation domain derived from patient’s anatomical imaging. Colors indicate separate tissue types or collections of tissues that were assigned specific mechanical material properties as indicated in Table 5.

**Figure 6:**
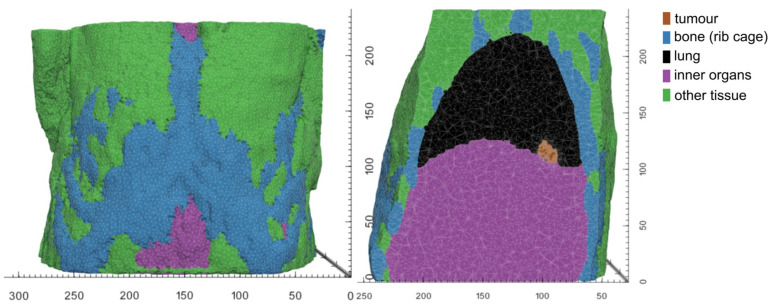
Personalized FEM model for NSCLC scenario with patient-specific FEM simulation domain, derived from patient’s anatomical imaging. Colors indicate separate tissue types or collections of tissues that were assigned specific mechanical material properties as indicated in Table 5.

While material parameters and boundary conditions are assumed identical across patients within a simulation scenario, differences in patient anatomy are accounted for by solving the equations of continuum elasticity on a patient-specific domain. These personalized computational models are created by first segmenting the region and tissues from clinical imaging of each patient. From the segmentations of each patient’s anatomy, tetrahedral meshes are generated using an in-house C++ tool based on open-source libraries. Figure 5 and Figure 6 illustrate patient-specific BMS simulation domains for one exemplary patient of the WT and NSCLC scenario, respectively.

## 3. Results

### 3.1. Assessing Personalized Predictions for NSCLC Adenocarcinoma: A Proof of Concept Study

A 63-year-old female patient with a past history of lung cancer is considered in the present study. The patient was followed up and treated at the Institute of Pathology of the University Hospital of Saarland. The study involves the progression and response to radiation treatment of a cancer recurrence that the patient developed.

Treatment schedule considered: The patient received external radiation of the right upper lobe. Four fractions of 15 Gy were given, once a day, three days/week. The radiation schedule considered is detailed in Appendix C.

Patient-specific data: Histological examination of the resected primary section revealed NSCLC adenocarcinoma of stage IB disease (pT2aN0M0) (TNM Classification of Malignant Tumors, 7th ed.) with acinar growth patterns (grade II). The proliferative index determined by Ki-67 labeling was 23%. Mutation analysis revealed the presence of the KRAS mutation Gly12Cys, but no EGFR, BRAF, ALK or ROS-1 alterations. Furthermore, tumor and normal lung tissue samples were analysed for the expression levels of 2549 miRNAs. Values are considered in the present study using quantile normalization [51].

Approximately three years after surgery, successive CT scans show the appearance and progression of a recurrence. Two CT imaging sets of the recurrent cancer acquired three months and one week before the onset of radiotherapy are available for study purposes. A follow-up with a CT scan one year after irradiation revealed no tumor presence in the treated area.

Due to the non-availability of biopsy-related data, as a first approximation, the mutation data, miRNA expression values, and the Ki-67 proliferation index of the recurrent cancer are considered the same as the ones of the primary tumor (time point T0). The hypermodel also considers the applied radiotherapeutic scheme (dose, radiation instants), and the 3D image of the tumor as reconstructed from the segmented CT imaging data. In the absence of volumetric data that allow the delineation of any tumor metabolic subregions, segmentation has been restricted to the boundary of the tumor. Hence, the virtual tumor is assumed homogeneous with a shape compliant to the reconstructed tumor image.

Predicted cell kill rate: The predicted cell kill rate from the molecular model was compared with that obtained from the empirical LQ model (Table 6). We observe that the predicted cell kill rates converge at higher radiation dosages but molecular model predictions are lower compared to the LQ model at lower dosage fractions. The results obtained from the molecular model are averages over various growth factor and time scale considerations taking into consideration the molecular profile of the patient.

Predicted treatment outcome: The clinical questions addressed by the hypermodel concern the prediction of tumor recurrence and in the case of recurrence, to predict the volume of the tumor one year following the completion of tumor irradiation. The progression ‘phase’ of the recurrent tumor before irradiation is used to adapt the Lung Oncosimulator. More specifically, a group of virtual tumors that constitute solutions to the same adaptation problem and efficiently cover the parameter space are derived. The following proliferation constraints/assumptions have been exploited: (a) the virtual tumor implementations must have a growth fraction (*GF*) equal to the proliferation index (Ki-67) of the patient (=0.23), (b) the volume doubling time must be around 370 days and (c) the population composition should be within the value ranges reported in Table 7. Furthermore, the ranges of the model parameters considered are given in Table 7. The doubling time has been estimated based on the observed volume increase between the two available volumetric data before the radiation therapy. In the second step, the Oncosimulator is run to simulate tumor progression and treatment response and predict recurrence and tumor volume after irradiation.

LHS has been run to generate 200 combinations of parameter values that fulfill the above requirements, following the methodology described in Section 2.3.2. Combinations that result in biologically non-relevant tumors e.g., negative cell class transition rates *P_sleep_* and *R_NDiff_*, or in tumors with non-relevant proliferation dynamics e.g., stem cell fractions out of range, are excluded.

The first clinical question is addressed by computing the tumor control probability (TCP), which is the probability that no clonogens survive after treatment [72]. We have adopted the Poisson model of TCP, which is considered a good approximation when the surviving fraction is <<1 [72,73], as in our clinical case: TCP = exp(−*N*), where *N* is the average number of surviving clonogens or cancer stem cells (CSCs) at the end of treatment. In our case, N is derived based on the execution of the hypermodel. In particular, because the Oncosimulator explicitly models the proliferation and treatment-induced death of CSCs, the number of CSCs that remains at the end of the radiation treatment can be computed for each virtual tumor. The number of remaining CSCs depends on their initial number. The latter is determined based on initial tumor volume, assumed tumor cell density (10^6^/mm^3^) and the value of OS input parameters related to the kinetics of CSCs. Proper adjustment of the considered value ranges ensures that the fraction of CSCs is within the range of TIC (tumor-initiating cells) frequency reported in the literature (Table 7) for the majority of the virtual tumors returned by the LHS. Virtual tumors having an initial frequency of cancer stem cells beyond this range are excluded from the analysis.

Three scenarios are demonstrated here. In all scenarios, the cell kill rate of cells in all phases is considered equal to the estimation of the molecular component for the specific patient and the radiation dose considered. Moreover, the withdrawal of cells in a quiescent phase, as a means to adapt to the local nutrient (glucose) conditions, is regulated by the vasculature and metabolic components. A sufficient average vessel density and glucose consumption rate are considered because the presence or extent of necrosis, which is associated with the local disappearance of blood vessels, is usually low in this histological type [69,70]. The first scenario considers the dose that was actually administered (15 Gy), while the second scenario corresponds to a lower radiation dose (10 Gy). In the third scenario, radiation therapy is given one month earlier.

**Figure 7:**
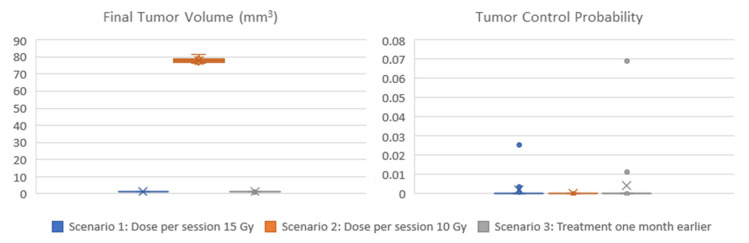
Box-and-whisker plots of the predicted volume one year post radiotherapy and the estimated TCP for a NSCLC case. Scenario 1 considers the applied therapeutic scheme and dosage. Scenario 2 assumes a reduced administration dose. Scenario 3 assumes that radiotherapy is administrated one month earlier.

Figure 7 displays the box and whisker plots of the estimated TCP and the predicted tumor volume one year following radiotherapy for the three clinical scenarios. In the first scenario that exploits all available imaging, treatment, and molecular data, a TCP close to zero is estimated (median TCP: 4 × 10^−12^, IQR: 2 × 10^−22^–7 × 10^−5^), suggesting that the tumor will recur. The volume of the predicted lesion is approximately 0.91 mm^3^ (median: 0.908, IQR: 0.909–0.912) at the time point of the final CT acquisition. The predicted volume size is below the detection limit [74] for all virtual tumors implemented. Administration of a lower dose per radiotherapy session (scenario 2) would result again in no local control (TCP: 0), while the predicted tumor size is much larger (median: 78 mm^3^, IQR: 77–79 mm^3^). If radiotherapy would be given one month earlier (scenario 3), TCP would not improve (median: 7 × 10^−12^, IQR: 7 × 10^−20^–9 × 10^−6^).

Summarizing, the hypermodel predicts (scenario 1) a tumor of an equivalent diameter of approximately 0.97 mm i.e., a tumor not easily detected. Based on patient data no visible tumor exists one year after irradiation. Even though hypermodel predictions seem consistent with reality, follow-up data beyond this period would be needed to properly validate the hypermodel, for the specific clinical case.

### 3.2. Clinical Adaptation and Partial Validation of the Hypermodel: A Proof of Principle Study for Wilms Tumor

Two clinical cases of Wilms tumor have been selected for the present study. The patients were diagnosed and treated at the Department of Pediatric Oncology and Hematology of the University Hospital of Saarland. The study involved the response to combination chemotherapy.

Treatment schedules considered: Both patients received preoperative chemotherapy with a 4-week regimen of vincristine (1.5 mg/m^2^, maximum 2 mg) and actinomycin D (45 mg/kg IV, maximum 2 mg) according to the SIOP 2001/GPOH clinical trial for unilateral stage I-III nephroblastoma tumors. (Appendix C). For Case 1, only a schedule was available. Dosage was assumed based on other patients.

Patient-specific data: Because of the fragile nature of Wilms tumor, no biopsy is performed in clinical practice and the diagnosis is always made after the surgery. For the cases considered, the histological reports of the resected tumors were not available.

The hypermodel considers the normalized serum miRNA data, the applied chemotherapeutic scheme (dose, administration times), and the 3D image of the tumor as reconstructed from the segmented MRI imaging data. Two MRI imaging sets of the tumor acquired before and after chemotherapy are available for the study purposes. Because of the lack of macroscopically distinct tumor subregions, the virtual tumor is assumed homogeneous with a shape compliant to the reconstructed tumor image.

Predicted cell kill rate: The cell kill rates predicted by the molecular model based on the normalized miRNA data are depicted in Table 8. Molecular data model a moderate response to combined chemotherapy for Case 2, while a high *CKR* is computed for Case 1.

Assessment of proliferation profile: The hypermodel is applied to estimate the proliferation profile of the examined clinical cases. The following tumor-proliferation features have been considered based on the literature: a. volume doubling time: *T_d_* = 11 days, 25 days, 40 days, b. growth fraction: *GF* = 10%, 25%, 50% and c. cell proliferation times = 13.1 h, 20 h, 50 h corresponding to high, moderate, and very low glucose concentration (Figure 4a), leading to 27 proliferation profiles i.e., pairs of (*T_d_*, *GF*, cell proliferation time). The value range of the input parameters is reported in Table 9. Only parameters related to free growth are varied. Cell-kill rates are fixed to the patient-specific estimates from the molecular model (Table 8). LHS has been run to generate *60* virtual tumors (combinations of parameter values) for each pair of (*T_d_*, *GF*, cell proliferation time). Combinations that result in negative cell class transition rates, namely negative *P_sleep_* and *R_ADiff_*, are excluded. For each virtual tumor, the Oncosimulator simulates the therapeutic plan of each clinical case (Appendix C) and the treatment-induced volume reduction is predicted. The real chemotherapy-induced shrinkage of tumor volume is compared against the predicted volume reduction to determine the proliferation profiles that are compatible with each clinical case.

**Figure 8:**
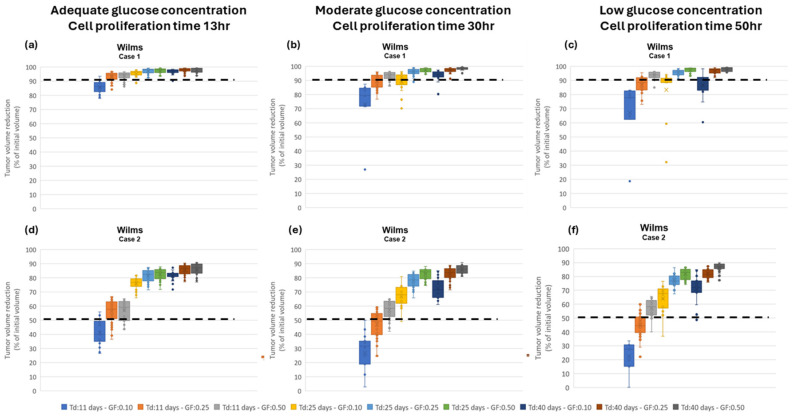
Box-and-whisker plots of the predicted volume reductions at the time of 2nd image acquisition for two WT patients. Black dashed line corresponds to real volume reduction. 9 proliferation profiles, i.e., combinations of (*T_d_*, *GF*), and 3 cell proliferation times have been considered. Based on metabolic model the cell proliferation time of WT cancer cells can vary between approximately 13 and 50 h depending on glucose concentration (Figure 4a). Panels (**a**,**d**) correspond to an adequate glucose concentration and a low cell proliferation time = 13 h. Panels (**b**,**e**) correspond to a moderate glucose concentration and a cell proliferation time = 30 h. Panels (**c**,**f**) correspond to a low glucose concentration and a cell proliferation time = 50 h.

The boxplot of the predicted volume reductions for each combination of *T_d_*, *GF*, and cell proliferation time is depicted in Figure 8. The tumor volume doubling times cover the entire value range reported in the literature. The growth fractions chosen approximately correspond to median values for different histological types of WT [79,80]. The results clearly demonstrate the potential of the integrative hypermodel to predict tumor shrinkage following proper adaptation. In both cases, there are proliferation profiles that are consistent with the observed tumor behavior. For case 1 most virtual tumors suggest a high tumor shrinkage. In case 2 proliferation profiles not consistent with the observed behavior are evident. It is noted that for the specific predictions, the only personalized data utilized that could affect the predicted outcome were the serum miRNA expression data. They were used by the molecular model to assess chemosensitivity. The rest of the hypomodels utilized cancer-specific knowledge. The results demonstrate that the increased chemosensitivity of case 1 was successfully captured. Studies of this type can be used to link proliferation activity with response taking into consideration the sensitivity profile of the patient to therapy.

### 3.3. Assessing Evolution of Tumor Shape and Position

Available clinical medical images at two time points (*t_1_*, *t_2_*) were registered using a rigid registration procedure in order to establish a common spatial reference frame, facilitating comparison and analysis. Then, the position of the center-of-mass (COM) was computed for both images at the initial time point (*t_1_*), the second time point (*t_2_*), and at the various simulation timesteps *t_s,i_* between *t_1_* and *t_2_*. The spatial agreement between the simulation and reality is assessed by measuring the distance between the tumor center-of-mass positions of the simulated tumor at each simulation time step *t_s,i_* and the center of mass at the final imaging time point (*t_2_*). This distance metric serves as a measure of how well the simulation aligns with the actual imaging data.

**Figure 9:**
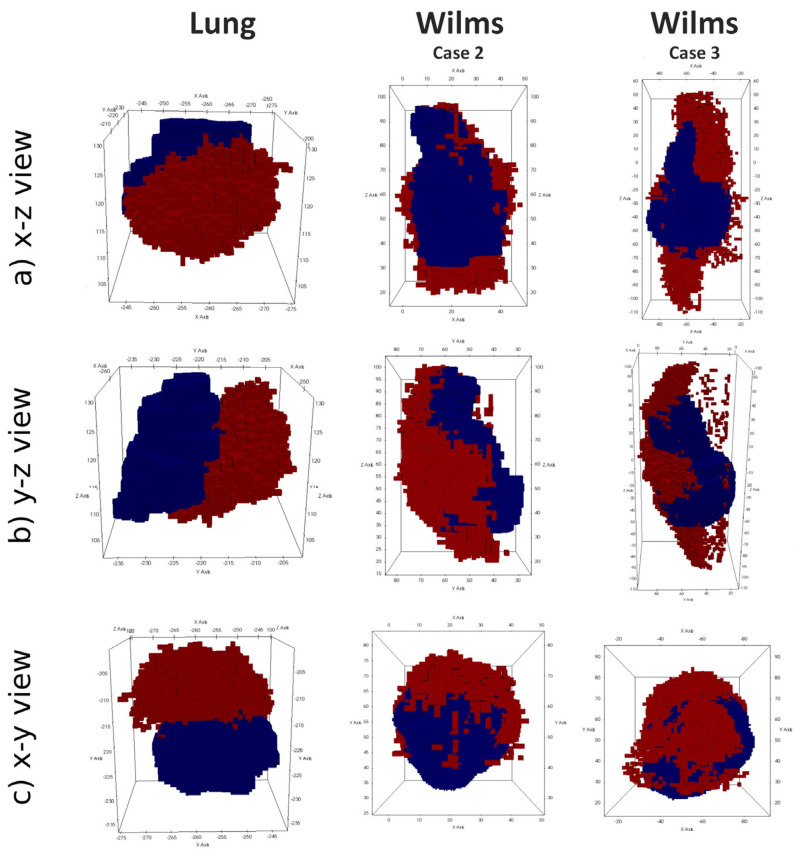
Visual comparison of shape and position between simulated (red) and observed (blue) tumors at the respective second imaging time point (*t_2_*). In the NSCLC scenario, during the simulation period (Days 1–98), the tumor volume increases (simulation of free growth before start of irradiation). In the WT scenarios, the tumors decrease (simulation of tumor response to chemotherapy) during the simulation periods (Case 2: Days 0–28; Case 3: Days 0–41).

This assessment strategy was applied to the results of the fully integrated WT and NSCLC hypermodels. Figure 9 illustrates the 3D shape and position of the simulated tumor in comparison to the actually observed tumor. For the lung scenario medical clinical images at the time of diagnosis (*t_1_*) and after three months of free growth (*t_2_*) were acquired. For the WT scenarios, medical imaging was acquired at the time of diagnosis (*t_1_*) and after the completion of the administered chemotherapy scheme (*t_2_*). During the simulation period, tumor volume increases in the NSCLC scenario and decreases in the WT scenario. The simulated free-growing tumor in the NSCLC scenario maintains a compact shape, in agreement with observation. Its simulated and observed positions at the second imaging time point are approximately 2 cm apart. Likewise, the COM distance remains in the range of about 2 cm for the two selected WT cases. Visual comparison of tumor shape shows that the simulated tumor does not shrink isotropically to a compact bulk tumor with a smaller radius as expected from the segmentations of the second imaging time points. Instead, the tumors appear to dissolve from one side, forming a porous and partially disconnected structure.

## 4. Discussion

The presented multi-scale hypermodeling framework combines subcellular processes related to cell proliferation and cellular response to therapeutic agents, as well as macroscopic processes such as biomechanical interaction between healthy tissue and tumor. Two cancer types, Wilms tumor and non-small cell lung cancer, were addressed, considering chemotherapy and radiation therapy as treatment modalities. The application of this framework aimed to address different clinical questions related to tumor shrinkage after neoadjuvant therapy and tumor recurrence.

The selection of cancer types and clinical scenarios is based on their capacity to map and tackle issues related to the response to preoperative therapy or recurrence following non-surgical treatment. Roughly 7% of malignant pediatric tumors are renal tumors, with nephroblastoma or Wilms tumors (WT) accounting for approximately 90% of these cases [85]. The WT is ideal for the construction and validation of the spatiotemporal hypermodel due to the consistent administration of chemotherapy before surgery in all pediatric patients, coupled with regular monitoring utilizing 3D imaging modalities both pre- and post-chemotherapy. However, around 10–20% of patients do not respond to pre-operative chemotherapy [86,87]. For these patients, primary surgery would be beneficial. Therefore, a primary clinical question that the hypermodel could answer is the following: Will a given nephroblastoma in a patient respond to pre-operative chemotherapy by tumor shrinkage, yes or no? Moreover, accurate prediction of tumor localization after chemo is relevant for surgical planning, particularly in procedures like nephron-sparing surgery. Knowledge of vascular pathways and potential adherence to other organs like liver, spleen, pancreas and colon is vital for optimizing patient outcomes and minimizing risks. Finally, multiparametric analyses [14,19], as well as the proof-of-concept studies presented in the present work (Section 3.2), reveal that the tumor shrinkage after chemotherapy is not only influenced by the sensitivity of tumor cells to the drugs administrated but depends largely on the proliferation profile of the tumor. The histology and proliferation index of WT at the time of diagnosis is unknown because no biopsy takes place. An indirect way of determining them would be of paramount importance in order for the clinician to judge whether or not a particular patient would benefit from chemotherapy. miRNA pattern from serum and blood at the time of diagnosis may be used as a surrogate indicator of the actual cell type composition of the tumor and its proliferation characteristics. Machine learning can be recruited to link the in vivo proliferation estimates, as attempted in the present work, with the serum miRNA profiling of a patient. The proliferation estimates in the present work were provided based on the chemo-induced tumor shrinkage measured from medical images, considering the sensitivity profile of the patient to therapy according to the output of the molecular model.

Lung cancer ranks as the second most prevalent form of cancer globally and the primary contributor to cancer-related death [88]. Overall, the 5-year survival rate is low, amounting to approximately 20% [89]. The poor survival is attributed to resistance to treatment and local or distant relapses. In the case of radiotherapy, the major treatment of NSCLC, hypoxia due to disorganized vasculature, cancer stem cells and mutational status (e.g., EGRF, KRAS, etc.) are believed to be among the key factors in resistance [90]. The management of local recurrences also remains challenging [91,92]. It may involve radiotherapy re-treatment using conventional or advanced techniques (i.e., intensity modulated radiation therapy, stereotactic body radiation therapy, proton beam therapy), chemotherapy, targeted therapy, or surgery, depending on the stage and previous treatment. Re-irradiation poses risks of severe toxicity for previously irradiated critical organs and consensus on the optimal re-irradiation dose is lacking [91,93]. Moreover, the efficacy of combining re-irradiation with systemic treatments, like chemotherapy, and the ideal delivery sequence (concurrent or sequential) remain uncertain [93]. The hypermodel serves as a powerful tool for the analysis of the combined effect of signaling networks, particularly those implicated in treatment resistance, alongside other resistance mechanisms. Furthermore, it has the capability to address clinical questions related to the management of inoperable primary tumors or recurrences. The hypermodel plays a crucial role in evaluating tumor control probability by assessing surviving clonogens under different treatment approaches, aiding in the selection of the most suitable strategy to prolong survival. Early detection of recurrences is vital for better clinical prognosis [94], especially in lung cases where radiological assessments alone may not adequately discern small lesions, such as those up to 3 mm in size. These limitations of imaging modalities in detecting such lesions emphasize the need to integrate approaches for accurate lesion characterization and timely differentiation between tumors and other conditions, such as infections, scars or post-treatment changes. In instances where local recurrence is anticipated, the hypermodel can assess when the tumor is expected to become clinically detectable, facilitating more effective patient follow-up.

The results are relevant to the specific cancer types. However, the methodology itself can be adapted, and, more importantly, the models can be adapted and trained to additional cancer types and clinical questions, leading to further reusability and extensibility of the overall framework.

Finally, traditional therapeutic advancements in clinical settings predominantly rely on randomized clinical trials, which aim to identify favorable treatment outcomes on average. However, patient responses to therapies often vary significantly from this average behavior. Integrated approaches like the ones presented here can be of great clinical value in determining drug effectiveness, dosage, and duration, as well as investigating the development of resistance to drugs and the effect of intra- and inter-tumoral heterogeneity. Multiscale cancer modeling holds promise in elucidating why certain treatments fail while others effectively control tumor progression, as well as why a specific therapy is effective only in a subset of patients. Eventually, by training the model using individual patient data, a more precise depiction of disease progression kinetics can be attained.

At the molecular level, the p53-mediated signaling pathways are particularly important in determining tumor cell response to DNA damage chemotherapeutic drugs like doxorubicin and vincristine as well as radiation therapy [95]. In the present work, we presented an integrated molecular model to model key cell signaling pathways operating at different time scales—a well-recognized challenge in the field. We model the p53-mediated DNA damage-response pathway, and we refine its predictions by running a model of the ErbB receptor-mediated Ras-MAPK and PI3K/AKT pathways. Information is passed across the identified interfaces in both directions. In order to consider the effect of patient-specific molecular profiling, we have also incorporated the miRNA expression and various mutation data to renormalize the initial expression levels of corresponding mRNAs to a particular patient. In doing so, we have also taken into account the heterogeneity of the microenvironment and have adopted an ensemble of models approach by averaging over multiple conditions of receptor expression, growth factor availability, and the nature of the memory coupling signaling and transcriptional modules. The aim is to provide a mechanistic foundation to the more empirical models to obtain patient-specific cell kill rates under particular dosage conditions.

The obtained cell kill rate was directly incorporated as an input to the Oncosimulator. The Oncosimulator serves as an integrator, effectively bridging scales and facilitating the “exposure” of molecular mechanisms to the scale where the outcome is formulated. The Oncosimulator is built based on the cancer stem cell hypothesis and accounts for tumor repopulation during and after treatment assuming different tumor proliferation dynamics and varying degrees of adaptation to nutrient-deprived conditions. As the tumor grows well-vascularized regions providing sufficient nutrients to cancer cells can coexist with nutrient-limited regions within the tumor mass. In this work, glucose is assumed to be the only limiting resource, although oxygen can also be incorporated as well as glutamine. The metabolic component models the dependence of glucose uptake on glucose concentration, using Michaelis–Menten kinetics at the genome scale. The model encapsulates the metabolic adaptations exhibited by highly proliferating human cancer cells and provides information to the Oncosimulator regarding the cell proliferation rate given the available glucose. The model is developed by utilizing Recon1, the first human Genome-Scale Metabolic Model (GSMM) and constraining certain metabolic fluxes in a simplified manner. Simulations suggest that the model adequately mirrors the glycolytic phenotype, showing increased growth rates, elevated lactate production, and a decline in growth yield with escalating glucose concentrations. The predicted cellular growth rates align with the characteristics observed in the studied cancer types. Moving forward, we aim to further refine the model through advancing algorithms for generating GSMMs specific to cancer cell lines and tumors. Additionally, we plan to develop patient-specific metabolic models by conducting transcriptomic profiles of biospecimens at tissue and cellular resolution and performing in vitro experiments utilizing patient-derived cancer cells. For such in vitro experiments, it is necessary to define a more physiologically relevant environment, including nutrients such as BCAAs, fatty acids, and glucose, to better mimic human blood and the tumor metabolic microenvironment. Through these efforts, we aim to enhance the predictive capacity of our models, rendering them more reflective of the intricate patient-specific metabolic landscape of cancer.

The local concentration of glucose is described by the vasculature model. A simple vasculature model was constructed as the first to be used in the development, verification, and validation of the WT and NSCLC multi-modeler hypermodels. More detailed models, as described subsequently, can be readily incorporated into the framework if justified by available clinical data. First of all, several ‘nutrient’ fields can be considered such as oxygen and glutamine. In reality, when used to model glucose transport, oxygen availability should also be accounted for, as per [50]. Another aspect is that the metabolic hypomodel uses an independent model of glucose consumption. In theory, the rate of glucose consumption from the metabolic model could be passed back to the vasculature component and used to update glucose concentrations. Moreover, the vasculature is assumed to be ‘static’ in the current model, in that it does not evolve in time. If justified by available data temporal evolution of the vasculature can be easily included. In addition to the tissue-scale transport model used in the clinical demonstrators, a range of more spatially resolved models of transport in tumor micro-vessels have been developed using Chaste [26]. These models can be used to inform the hypomodel used in the present work.

The hypermodel is found to reproduce realistic tumor shapes in growth scenarios (Lung), whereas shrinkage scenarios (Nephroblastoma (WT)) tend to result in tumor shapes that have a more ‘diffuse’ appearance than those observed. The hypermodel achieved a good prediction of tumor position in the simulated cases. The following limitations may explain the observed discrepancies and could be the subject of future research. A critical issue for the Biomechanics Simulator is the uncertainty in mechanical tissue parameters (not patient-specific) and the lack of well-defined boundary conditions for mechanical computations that are particularly difficult to establish for the WT and Lung scenarios. Furthermore, the evaluation relies on image registration techniques to compare simulation results to imaging data at a later time point. This process introduces an uncertainty in the relative positioning of the tumors. To reduce the importance of this uncertainty in future studies, the use of fixed anatomical markers as reference within the respective imaging frame could be investigated. Another limitation is related to the complexity of the coupling between OS and BMS. Mapping of the pressure (direction of least-pressure) field computed by BMS into the discrete model of OS is challenging, as is the update of BMS with OS cell concentration values. Accuracy in both steps is affected by interpolation. Mesh creation from image segmentation and mapping of 3D parameter distributions between domains are commonly used in Finite Element or Finite Difference-based simulations. These “convenience functions” are crucial for functioning simulator components. We believe that each of these functionalities could be well encapsulated in a standalone hypo-model in the future. This would not only greatly facilitate the creation of new personalized FEM models and the parameter exchange between other component simulators; it would also ensure consistent handling of these critical simulation and communication aspects across the platform. Finally, morphological changes in the healthy tissue also influence tumor evolution. This aspect is not taken into account by the present hypermodels.

It is pointed out that the models proposed/developed originates from a rather macroscopic approach to tumor response to treatment as was adopted by classical radiobiology. Several steps have been made in order to go deeper and deeper into microscopic mechanisms. But due to the great complexity of cancer mechanisms up to now not all possible factors have been considered such as the microenvironment or the immune system, nevertheless this can be done in a way pretty similar to the one adopted through the use of hypomodels so far. That means that additional hypomodels each one representing a not yet addressed factor of phenomenon can be developed and linked to the core of the oncosimulator.

Before using a hypermodel in clinical settings, it needs to be clinically validated to ensure that it is accurate and reliable. It should occur at the levels of both the hypomodels and the hypermodel. Validation usually follows a 2-step approach, in which first the model has to be calibrated to a specific patient using information from an early observation point, and second, the model’s predictions about a later observation point are compared to the actual disease evolution. For example, in the case of the BMS, the first step involves the creation of a patient-specific simulation domain, while in the case of the oncosimulator, a cohort of virtual tumors is created specific to the proliferation characteristics of patients’ tumors (e.g., Ki-67, etc.). The next step involves a forward simulation of the calibrated model to a later time point and a comparison of the simulation results to the patient’s disease evolution, e.g., in terms of tumor location and shape as presented in this manuscript or tumor volume reduction. Approaches for recovering important parameters for biomechanically coupled tumor growth models from single observation points have been investigated in [96]. Furthermore, in vitro data and experiments can serve as valuable tools for the calibration and preliminary validation of a hypomodel. For example, by conducting experiments in vitro based on patient-derived cancer cells and testing against experimental data, cancer- and patient-specific metabolic models can be built, as previously discussed.

The clinical validation of the hypermodel should initially be conducted using retrospective data from datasets distinct from those utilized for clinical adaptation and calibration. These datasets may originate from the same or other clinical studies, the latter ensuring robustness and generalizability of the model’s performance. Following sufficient preliminary clinical validation, the next step would be to conduct a prospective blinded clinical trial. This trial aims to investigate whether utilizing the hypermodel’s predictions correlates with improved treatment outcomes compared to standard approaches that do not incorporate the hypermodel. For example, a hypermodel indicates whether a preoperative chemotherapy scheme is better than primary surgery. One should look on tumor volume reduction predicted by the hypermodel. If the hypermodel predicts a reduction, chemotherapy is selected; if the hypermodel does not predict a reduction, go to primary surgery. In the standard arm, all patients will receive preoperative chemotherapy. Comparing the results between the two arms would reveal whether the model is beneficial for the outcome of a patient. When utilizing retrospective data or for prospective trials consisting of one standard arm, one could compare the reduction in tumor volume with the predicted reduction by the hypermodel as a validation means of the hypermodel. Currently, there is an ongoing validation effort concerning nephroblastoma within the context of SIOP (International Society of Paediatric Oncology) clinical protocols. A new infrastructure has been established within the University Hospital of Saarland to facilitate the collection, storage, retrieval, curation, and utilization of multiscale data generated during nephroblastoma treatment. Additionally, this infrastructure is capable of executing the multiscale mechanistic simulation models comprising the Nephroblastoma Oncosimulator directly within the hospital environment.

The potential benefits of implementing a hypermodel in a clinical setting include improved accuracy and efficiency in treatment selection and prognosis prediction, leading to better patient outcomes and enhanced quality of care. Hypermodels can enable personalized medicine by tailoring treatments to individual patient characteristics, thereby maximizing therapeutic efficacy and minimizing side effects, e.g., by avoiding ineffective pre-surgery chemotherapeutic treatments for certain patients. Moreover, hypermodels can also help healthcare providers optimize resource allocation, reduce healthcare costs, and streamline workflows by automating repetitive tasks or providing decision support.

However, integrating a hypermodel in a clinical setting presents several challenges related to the familiarization of clinical doctors with the new technologies necessary for the exploitation of the hypermodels, data privacy, security, and integrity as well as increasing computer power and memory.

Integrating the model into existing workflows without disrupting clinical operations or overwhelming healthcare providers with additional information or tasks is important [97]. Seamless adoption and effective utilization may involve developing interfaces or APIs for data exchange between the hypermodel and the clinical workflow software [97]. Resistance to change among healthcare professionals, skepticism about the utility of the model, and concerns about job displacement due to automation are common barriers to successful implementation. Ensuring the hypermodel’s reliability is of outmost importance for clinicians to accept it, as incorrect clinical decisions based on inaccurate predictions could potentially harm the patients. Training healthcare professionals on how to use the hypermodel is crucial for successful integration. Providing user-friendly interfaces and clear guidelines for incorporating the hypermodel’s predictions into clinical decision-making processes can facilitate adoption among clinicians. The following example can serve as a model for a generalized guide for the integration of hypermodels into existing clinical workflows. In the case of nephroblastoma, there are two initial treatment approach options. To either proceed to the neoadjuvant chemotherapeutic treatment and then proceed to the surgical excision of the tumor, or to start with the surgical excision of the tumor and administer chemotherapy afterwards. A relevant hypermodel could predict whether or not the shrinkage of the tumor due to neoadjuvant chemotherapy would be greater than a minimal clinically acceptable threshold (e.g., 30% reduction in the sum of lesion diameters based on imaging studies [98]). In such a case neoadjuvant chemotherapy is applied. This will lead to a considerable shrinkage of the tumor and therefore a smaller surgical field in the surgery to follow. Otherwise, surgery takes place since neoadjuvant chemotherapy would not essentially shrink the tumor, whereas it will only create side effects.

Personalized simulations and virtual digital twins may potentially raise ethical implications related to data privacy, security, and integrity, as well as patient consent [99]. A multi-faceted approach is required to protect against a possible misuse of sensitive personal data or an unauthorized or accidental modification/deletion of data, to ensure confidence in model predictions and to maintain patient trust. Patient data should be subject to pertinent legislation, including the General Data Protection Regulation (GDPR) in European Union, Health Insurance Portability and Accountability Act (HIPAA) in the United States, and applicable national laws, as well as pertinent ethical guidelines as these are specified and approved by the clinical center ethical committee. A secured IT infrastructure should be implemented including firewalls, data encryption, and authentication and authorization mechanisms, required to guarantee a secure storage of data and models. Furthermore, data should be either anonymized or pseudonymized while transmitted. The data transmission can be secured using, for example, HTTPS and DICOM web protocols. Finally, patients should be informed about the possibility that their data are used for modeling and simulation purposes and they must fully comprehend how their data will be utilized. Any such use of their data should be made possible only if the patients have provided their written informed consent unless otherwise specified by the clinical center ethical committee.

Finally, creating and upkeeping virtual digital twins in healthcare necessitate computational resources, storage capabilities, and data-processing power [100]. The scalability issue emerges due to the vast amount of patient data involved in or produced by the model executions. Cloud-based infrastructures can offer dynamic adjustment of resources based on demand. Distributed computing architectures and data compression techniques can help optimize resource utilization. Standardized data formats can facilitate interoperability and scalability across different healthcare settings. Parallelization of model executions, e.g., concurrent execution of the virtual tumors across multiple processing units, combined with high-performance computing resources can reduce the overall execution time and can enhance scalability, particularly in scenarios where large datasets or complex algorithms are involved.

## 5. Conclusions

Based on the partial validation results and analyses that have been reported in this document, the highly innovative CHIC hypermodels and Oncosimulators appear to possess a great potential for serving as clinical decision support systems (CDS) and/or cores of future in silico trial platforms. However, additional retrospective validation work for the developed hypermodels and Oncosimulators is needed in order to fully substantiate and support their “candidacy” for undergoing validation through prospective clinical trials. This is a necessary step for assessing their clinical validity and clinical value. Further retrospective validation work will be carried out by specific former CHIC partners on a bilateral or small partner group basis. Regarding the eventual prospective clinical validation of the hypermodels, certain exploratory steps have already been taken, including focused discussions within the framework of the International Society for Paediatric Oncology (SIOP).

## Figures and Tables

**Table 1:** Model parameters of the Oncosimulator.

Symbol	Description	Units
**Cell phase durations**		
*T_c_*	Cell cycle duration	h
*T_G0_*	G0 (dormant phase) duration i.e., time interval before a dormant cell re-enters cell cycle or dies through necrosis	h
*T_A_*	Time needed for both apoptosis to be completed and its products to be removed from the tumor	h
*T_N_*	Time needed for both necrosis to be completed and its lysis products to be removed from the tumor	h
**Cell category/phase transition rates and fractions**		
*R_A_*	Apoptosis rate of living stem and limp tumor cells, i.e., fraction of cells dying through apoptosis per unit time	h^−1^
*R_ADiff_*	Apoptosis rate of differentiated tumor cells	h^−1^
*R_NDiff_*	Necrosis rate of differentiated tumor cells	h^−1^
*P_sym_*	Fraction of stem cells that perform symmetric division	-
*P_sleep_*	Fraction of cells entering the G0 phase following mitosis	-
*P_G0toG1_*	Fraction of dormant (stem and LIMP) cells that re-enter cell cycle	-
**Miscellaneous parameters**		
*N_LIMP_*	Number of mitoses performed by LIMP cells before becoming differentiated	-
**Radiotherapy—chemotherapy parameters**		
*CKR*	Cell kill rate: the numbers of biological cells lethally hit by treatment at each administration. In case of chemo, it is defined separately for each drug.	-
*α*/*β*	alpha to beta ratio	Gy
*D*	Dose of radiation to a population of cells	Gy

**Table 2:** Assumed parameter values for the metabolic hypomodel for WT and NSCLC.

Symbol	Description	Unit	Value	Source
$C_{max}$	Maximum glucose concentration	kg·m^−3^	0.9	[49]
$K_{m}$	Michaelis-Menten constant	kg·m^−3^	0.2704	[49]
*C_enz_*	Total enzyme mass	mg·(mgDW)^−1^	0.078	[10]
	Lower metabolic flux bound for reactions catalyzed by up-regulated genes	umol·(mgDW)^−1^·h^−1^	0.1	[35]
	Upper metabolic flux bound for reactions catalyzed by down-regulated genes		0	This work
	Glucose flux range	umol·(mgDW)^−1^·h^−1^	[0, 1.2]	[10]
	Lactate secretion rate tolerance	umol·(mgDW)^−1^·h^−1^	0.01	[35]

**Table 3:** Specific metabolism proteome clusters upregulated in lung cancer as reported in [47].

Cluster ID	Cluster Proteins (Gene Names)	Excluded from Model
C10	ADSS, ATP2A2, CTPS1, IMPDH2, PKM2, PTGES3, SGPL1	SGPL1
C15	NAT10, NME2, OAT, PPAT, SHMT2, GART, PAICS, SRM, UMPS, QARS, ABCE1, ABCF2, ACOT7	OAT, SHMT2

**Table 4:** Assumed parameter values for the vasculature hypomodel for WT and NSCLC.

Symbol	Description	Unit	Value	Source
D	Glucose Diffusivity	mm^2^·h^−1^	0.396	[50]
λ	Glucose Consumption Rate	(Num cells)^−1^·h^−1^	7.6 × 10^−10^	Modified from [50]
$c_{n}$	Glucose Concentration in Non Tumor Regions	kg·m^3^	0.9	Value used in metabolic hypomodel
ρV	Vascular Delivery Efficiency	h^−1^	0.25	User chosen/fit to data

**Table 5:** Tissues and mechanical tissue parameters for WT and NSCLC scenarios.

Tissue Type	E [Pa]	Poisson Ratio
**WT scenario**		
Healthy kidney	5.3 × 10^3^	0.40
Bone	1.0 × 10^9^	0.30
Other tissues	5.0 × 10^3^	0.40
Tumor	20.0 × 10^3^	0.40
**NSCLC scenario**		
Lung tissue	5.0 × 10^3^	0.40
Bone	1.0 × 10^9^	0.30
Other tissues	5.0 × 10^3^	0.40
Inner organs	5.0 × 10^3^	0.40
Bronchi	5.0 × 10^3^	0.40
Tumor	10.0 × 10^3^	0.40

**Table 6:** Cell kill rate (i.e., fraction of cells killed) averaged over various growth factor concentrations and cell cycle times as obtained from the molecular model compared with the results obtained from the LQ model. Typical radiosensitivity parameters are considered to be α = 0.35 Gy^−1^, β = 0.035 Gy^−2^ [52]. Values are rounded at the 3rd decimal place.

Dose per Fraction (Gy)	Cell Kill Rate Based on LQ	Modified Cell Kill Rate
5	0.928	0.433
10	0.999	0.712
15	1.000	0.905
20	1.000	0.979

**Table 7:** Assumed parameter values of the Oncosimulator and tumor characteristics for NSCLC.

Parameter	Value Range	Reference(s)
*T_c_* (h)	20–134	[53,54], the upper limit is constrained by the proliferation rate computed by the metabolic hypomodel
*T_G0_* (h)	96–240	[55]
*T_N_* (h)	1–100	[56,57], estimation based on the extent of necrosis reported in the literature
*T_A_* (h)	1–25	[58,59,60]
*N_LIMP_*	13–24	estimation based on frequency of tumor-initiating cells reported in literature
*R_A_* (h^−1^)	0–0.001	estimation
*R_ADiff_* (h^−1^)	0.0001–0.02	extension of [61,62,63,64]
*R_NDiff_* (h^−1^)	-	estimated per virtual tumor based on patient’s data (*GF*)
*P_G0toG1_*	0–0.2	
*P_sleep_*	-	estimated per virtual tumor based on the cell proliferation rate computed by the metabolic hypomodel
*P_sym_*	<0.3	[65,66], estimated per virtual tumor based on patient’s data (*T_d_*)
*CKR*	Study 1, 3: 0.905 Study 2: 0.712	estimated by molecular hypomodel based on patient’s miRNA and mutation data
*α* */* *β*	4–10	[67]
Cell proliferation rate	70 h	computed by metabolic model
Stem/living	0.00001–0.00025	[68]
*GF*	23%	Patient’s data: Ki-67
*T_d_*	370 days	patient’s data: tumor volumetric increase between two successive imaging data prior therapy
Necrotic/Total	<30%	based on [69,70]
Apoptotic/Total	<5%	[71]

**Table 8:** Cell kill rate (*CKR*) (i.e., fraction of cells killed) averaged over various growth factor concentrations and cell cycle times and adjusted to patient-specific genetic variation. Typical *CKR*s are considered to be *CKR_Vincristine_* = 0.28, *CKR_Actinomycin_* = 0.4, *CKR_combo_* = 0.568 (Calculated following methodology in supplement S3 from [14] based on data from [75,76]. Values are rounded at the 3rd decimal place.

	Vincristine (mg/m^2^)	Actinomycin (ug/kg)	Cell-Death-Mean Propability	*CKR* Adjusted
Case 1	0	0	0.179	-
	1	650	0.527	0.916
Case 2	0.83	0	0.112	0.28
	0.83	540	0.149	0.673

**Table 9:** Assumed parameter values for the Oncosimulator for the WT.

Parameter	Value Range	Reference(s)
*T_c_* (h)	11–50	[75,77,78], constrained by the output of metabolic hypomodel
*T_G0_* (h)	96–240	[55]
*T_N_* (h)	1–200	[56,57], estimation based on extent of necrosis in resected tumors reported in literature
*T_A_* (h)	1–25	[58,59,60]
*N_LIMP_*	13–24	Estimation based on frequency of tumor-initiating cells reported in literature
*R_A_* (h^−1^)	0–0.001	-
*R_ADiff_* (h^−1^)	0–0.02	-
*R_NDiff_* (h^−1^)	-	estimated per virtual tumor based on patient’s data (*GF*)
*P_G0toG1_*	0–0.2	
*P_sleep_*	-	estimated per-virtual tumor based on the cell proliferation rate computed by the metabolic hypomodel
*P_sym_*	-	[65,66], estimated per virtual tumor based on patient’s data (*T_d_*)
*CKR*	-	Estimated by molecular hypomodel based on patient’s miRNA
*GF*	0–80%	[79,80,81]
*T_d_*	11–40 days	[82,83]
Necrotic/Total	0–100%	[84]

**Figure A1:**
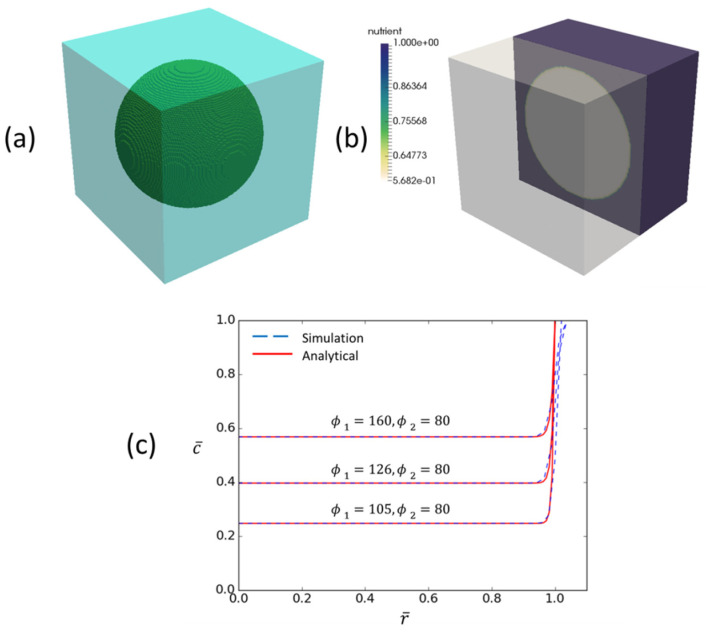
(**a**) The test geometry for the hypomodel, a 100 mm radius spherical tumor. (**b**) The predicted dimensionless nutrient field for $\phi_{1}=160$ and $\phi_{2}=80$. (**c**) A comparison of the hypomodel predictions versus Equation (A8). There is good agreement, within deviations at $\bar{r}=1$ due to the use of a regular grid to discretize the spherical tumor.

**Figure A2:**
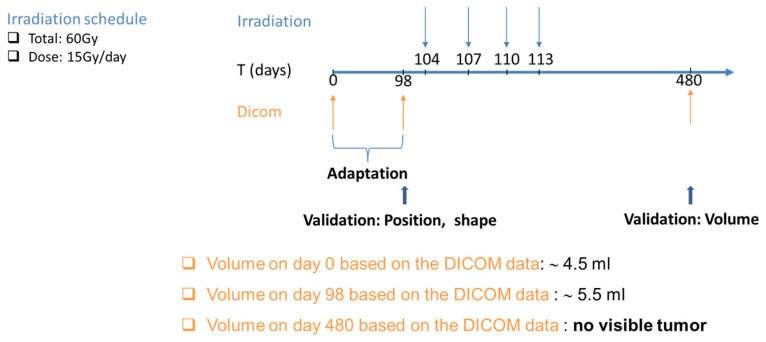
The simulation durations and chemotherapy protocols for the lung cancer case, presented in the paper (Section 3).

**Figure A3:**
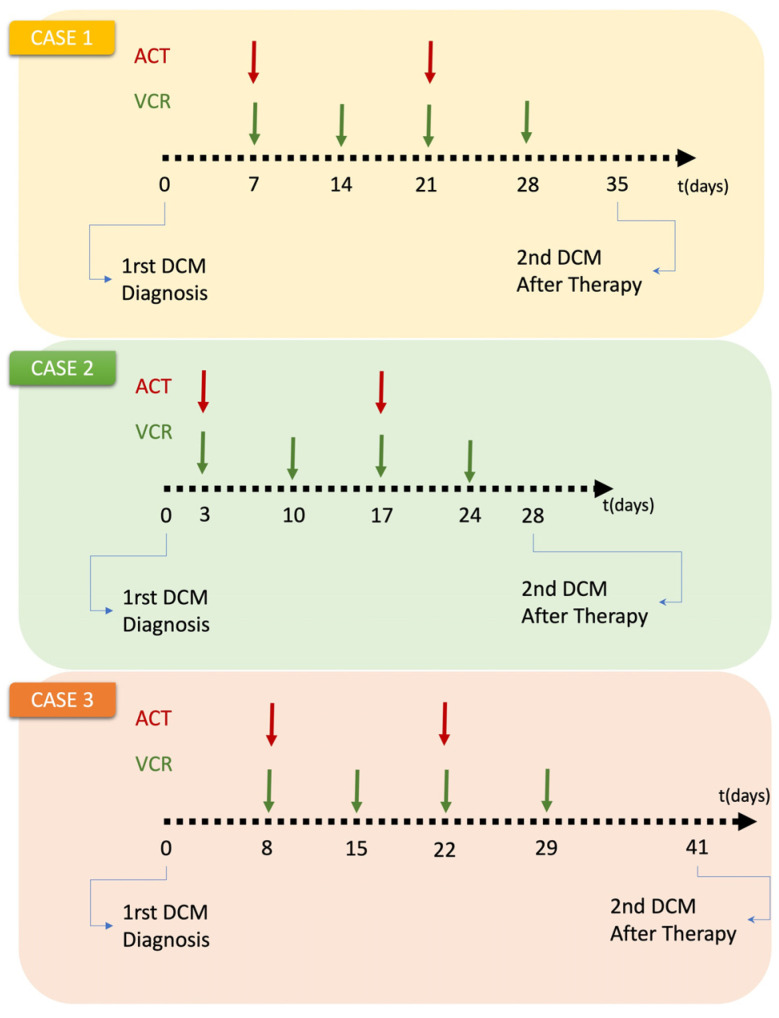
The simulation durations and chemotherapy protocols for Wilms cases, presented in the paper (Section 3).

## Data Availability

The core work of the manuscript was done during the implementation of the European Commission funded project CHIC (Computational Horizons In Cancer (CHIC): Developing Meta- and Hyper-Multiscale Models and Repositories for In Silico Oncology) that took place between 2013 and 2017 (https://cordis.europa.eu/project/id/600841, accessed on 1 March 2024). Since then, significant conceptual processing has taken place, especially in view of the current prioritization of the development and the clinical translation of digital twins, such as the CHIC oncosimulators and hypermodels. In this context, the multiscale clinical data used for the needs of the work reported ceased to be accessible one year after the completion of the CHIC project, in accordance with the consortium agreement and in line with the then applicable ethical and legal provisions, including GDPR. Meta-data are included in Appendix C. Regarding the computer codes, these have been developed and built in house by the modelling partners of the CHIC consortium and are not “open access”, with the exception of the vasculature hypomodel. It is worth mentioning, however, that the CHIC project outcomes were thoroughly reviewed and evaluated by external independent reviewers appointed by the European Commission. The CHIC project was evaluated by the independent reviewers as “excellent”, whereas its outcomes were designated as “great achievements” (https://digital-strategy.ec.europa.eu/en/news/great-achievements-eu-funded-chic-project, accessed on 1 March 2024).

## Appendix A

### Parametrization Methodology of the Oncosimulator

The Latin Hypercube Sampling method is used to generate a plausible collection of model parameter values that corresponds to virtual tumors having a common proliferation pattern in terms of volume doubling time, *T_d_*, and growth fraction (*GF*). We consider the fraction of newborn cells that enter the G0 phase, *P_sleep_*, and the necrosis rate of differentiated cells, *R_NDiff_*, as the dependent parameters of this multi-constrained problem. The independent variables comprise the rest of the model parameters that regulate tumor proliferation pattern, i.e., cell cycle time, *T_C_*, the duration of G0 phase, *T_G0_*, the duration of apoptosis, *T_A_*, and the duration of necrosis, *T_N_*, the apoptosis rate of stem and LIMP cells, *R_A_*, the apoptosis rate of differentiated cells, *R_ADiff_*, the symmetric division fraction, *P_sym_*, the fraction of stem cells that re-enter cell cycle from a quiescent state, *P_G0toG1_*, the number of mitoses performed by LIMP cells before becoming terminally differentiated *N_LIMP_*, and resistance of stem cells to chemotherapy, *CKF,* and the above-mentioned tumor proliferation features: *GF* and *T_d_*.

The analysis has been performed using the Matlab toolbox. The built-in function “lhsdesign” is run to produce N combinations of the independent model parameters: *T_C_*, *T_G0_*, *T_N_*, *T_A_*, *R_A_*, *R_ADiff_*, *P_G0toG1_*, *N_LIMP_*, *α*/*β*. LHS output is modified in the case of parameters with value ranges other than [0, 1] and parameters of integer type. For input parameters bounded in any range [*a_p_*, *b_p_*] other than [0, 1], the “lhsdesign” output has been rescaled by applying the formula:

(A1) $$a_{p}+\left(b_{p}-a_{p}\right)*x_{p},$$

where *x_p_* the vector of the *N* returned values for parameter *p*. For integer input parameters the rescaled values are rounded to the nearest integer.

For each set of the independent parameter values, parameter *P_sleep_* is computed based on the cell proliferation time returned by the metabolic hypomodel as described in Section 2.2.2.

Then, *P_sym_* and *R_NDiff_*, are derived from the following formulas, so as to achieve the given *T_d_* and GF.

Initially, the *P_sym_* value is computed so as to achieve the given *T_d_*: (derived from Equation (7) in [20])

(A2) $$P_{sym}=\frac{e^{(a+R_{A})Tc}}{1-P_{sleep}+P_{sleep}*\frac{P_{G0toG1}}{T_{G0}}/\left(a+R_{A}+\frac{1}{T_{G0}}\right)}-1,$$

where α = ln(2)/*T_d_*.

*R_NDiff_*, is calculated in order to achieve the particular *GF*: (derived from Equation (47) in S2 Text in [21])

(A3) $$R_{NDiff}=\frac{1-P_{sym}}{\frac{1}{A}\left(\frac{1}{GF}-1\right)-B}-a-R_{ADiff}$$

where

(A4) $$A=\frac{a+R_{A}}{e^{\left(a+R_{A}\right)T_{C}}-1}$$

(A5) $$B=\frac{1-P_{sym}}{a+R_{A}+\frac{1}{T_{G0}}}P_{sleep}$$

## Appendix B

### Validity Checks of Vasculature Component

An advantage of the simple nature of the adopted hypomodel is that it allows for testing and a clear path for model comparison with clinical datasets. This section overviews verification and testing of the final hypomodel implementation.

For testing it is useful to assume a spherical tumor of radius R. Non-dimensionalising Equation (4) with spatial coordinate $\bar{x}=xR$ and concentration $\bar{c}=cc_{n}$ gives:

(A6) $$\nabla^{2}\bar{c}-{\phi_{1}}^{2}\bar{c}+{\phi_{2}}^{2}=0$$

where $\phi_{2}=\sqrt{\frac{R^{2}}{D}\rho V}$ is a Thiele modulus related to vessel delivery efficiency and $\phi_{1}=\sqrt{\frac{R^{2}}{D}\left(\lambda P+\rho V\right)}$ is a Thiele modulus related to tumor consumption efficiency. Assuming a 100.0 mm radius tumor, the parameter values in Table 4, a typical cell number of 1 × 10^9^ in a region of interest and a vessel volume fraction of 1.0 gives $\phi_{1}$ = 160 and $\phi_{2}$ = 80, suggesting a reasonably high rate of glucose consumption versus delivery and very high reaction versus diffusion timescales. Converting Equation (A6) to spherical coordinates and dropping accents gives:

(A7) $$\frac{1}{r^{2}}\frac{d}{dr}\left(r^{2}\frac{d^{2}c}{dr^{2}}\right)-{\phi_{1}}^{2}c+{\phi_{2}}^{2}=0$$

with $\frac{dc}{dr}=0$ at r=0 and c=1 at r=1. This can be solved explicitly, giving

(A8) $$c\left(r\right)=\left(1-\frac{{\phi_{2}}^{2}}{{\phi_{1}}^{2}}\right)\frac{sinh\left(\phi_{1}r\right)}{rsinh\left(\phi_{1}\right)}+\frac{{\phi_{2}}^{2}}{{\phi_{1}}^{2}}$$

Solution values for a range of values of $\phi_{1}$ and $\phi_{2}$ are shown in Figure A1, along with hypomodel predictions. This serves as both hypomodel verification and a means for identifying suitable parameter values when attempting to describe clinical observations.

The following model serves as a simple method for relating the hypomodel inputs to tumor growth rate predictions using the dimensional form of Equation (A8), based on a more detailed treatment in [101]:

(A9) $$R^{2}\frac{dR}{dt}=\int_{0}^{R(t)}scr^{2}dr$$

where s is the tumor cell proliferation rate per unit nutrient concentration. Solution of Equation (A9) following the steps in [101] allows for the estimation of hypomodel parameter values from clinical data by fitting of clinically observed tumor growth rates. In the case of the demonstrator hypermodels and CHIC clinical data, more detailed, integrated, fitting is required.

## Appendix C

### Appendix C.1. Clinical Data of the Lung Case Studies Detailed in Section 3

For the Lung cancer case studies detailed in Section 3, three sets of MRI imaging data have been available, two at a time instant prior to chemotherapy and one approximately one year after the completion of radiootherapy. The clinical tumor volumes, as calculated based on the segmentation of the tumors on these imaging sets, are depicted in Figure A2 and Table A1.

The simulation duration and the exact treatment scheme administered for each, as derived from the available clinical data of the patients, are illustrated in Figure A2.

**Table A1:** Imaging data information of the lung cancer patient simulated and presented in Section 3.

Imaging	V_CT_ (cc)	DV_CT_ (%)
PRE 1st	4.5	22.22
PRE 2nd	5.5	

### Appendix C.2. Clinical Data of the Wilms Case Studies Detailed in Section 3

For the Wilms case studies detailed in Section 3, two sets of MRI imaging data have been available, one at a time instant prior to chemotherapy (*t_1_*) and one after the completion of chemotherapy and before surgery (*t_2_*). The clinical tumor volumes, as calculated based on the segmentation of the tumors on these imaging sets, are depicted in Table A2.

The simulation duration and the exact treatment scheme administered for each, as derived from the available clinical data of the patients, are illustrated in Figure A3.

**Table A2:** Imaging data information of the nephroblastoma patients simulated and presented in Section 3.

	Imaging	V_CT_ (cc)	DV_CT_ (%)
**Case 1**	PRE	78.54	90.68
	POST	7.32	
**Case 2**	PRE	109	52.29
	POST	52	
**Case 3**	PRE	754.75	80.43
	POST	147.68	
